# Computational strategic recruitment for representation and coverage studied in the *All of Us* Research Program

**DOI:** 10.1038/s41746-025-01804-x

**Published:** 2025-07-03

**Authors:** Victor A. Borza, Qingxia Chen, Ellen W. Clayton, Murat Kantarcioglu, Lina Sulieman, Yevgeniy Vorobeychik, Bradley A. Malin

**Affiliations:** 1 Department of Biomedical Informatics, Vanderbilt University Medical Center, Nashville, TN USA; 2 School of Medicine, Vanderbilt University, Nashville, TN USA; 3 Department of Biostatistics, Vanderbilt University Medical Center, Nashville, TN USA; 4 School of Law, Vanderbilt University, Nashville, TN USA; 5 Department of Health Policy, Vanderbilt University Medical Center, Nashville, TN USA; 6 Department of Pediatrics, Vanderbilt University Medical Center, Nashville, TN USA; 7 Department of Computer Science, Virginia Polytechnic Institute and State University, Blacksburg, VA USA; 8 Department of Computer Science, Washington University in St. Louis, St. Louis, MO USA; 9 Department of Computer Science, Vanderbilt University, Nashville, TN USA

**Keywords:** Clinical trial design, Databases, Data acquisition, Machine learning

## Abstract

Large scale data repositories like the *All of Us* Research Program are spurring new understanding of health and disease. *All of Us* aims to create a database of all Americans, addressing patterns of understudy of some groups in biomedical research. We study the representativeness (similarity to the U.S. population) and coverage (equality of proportion across U.S. Census demographic categories) of *All of Us* from 2017 to 2022, finding that *All of Us* recruited almost every understudied group at or above the group’s Census proportion. Building on the program’s successes, we propose a computational strategic recruitment method that optimizes multiple recruitment goals by allocating recruitment resources to sites and evaluate this method in recruitment simulation. We find that our methodology is indeed able to improve both cohort representativeness and coverage. Moreover, improvements in representativeness and coverage hold across numerous simulation conditions, supporting the promise of our recruitment techniques in real-world application.

## Introduction

Large biomedical datasets allow the discovery of groundbreaking new insights into health and disease. The need for, and utility of, these datasets will only increase with advances in artificial intelligence (AI). Yet, these datasets often do not adequately exemplify all groups in the population they are trying to study^1^. This understudying or underrepresentation occurs across many areas of biomedicine^2–9^, and can lead to several problems. For example, the results of scientific studies may not generalize to groups that are understudied or underrepresented in biomedical research (UBR), leading to an estimated economic cost of hundreds of billions of dollars in the United States from 2020 through 2050^10^. While many examples from that report concerned clinical trials and research into pharmaceutical efficacy, computational tools and techniques are similarly vulnerable to issues induced by lack of representation. Classifiers, a common type of AI tool, have sometimes been observed to be less accurate for groups underrepresented in their training data^11,12^. Unrepresentative datasets can skew statistical association measures leading to irreproducible results^13^. Lack of representation across studies may also impact the perception of research more broadly, leading to decreased trust and legitimacy^10,14^. Thus, ensuring adequate representation is critical.

Representation and coverage are abstract concepts, so assessing and improving them requires concrete measures. We follow common convention and define representation as a measure of similarity between a study cohort and some target population based on a set of attributes^15^. For this analysis, we measure the similarity of a cohort’s distribution of demographics (age, gender, race, and ethnicity) to the U.S. population’s true distribution of those same demographics, as determined by the Census Bureau. This approach has been used by others to assess the cohort representativeness of clinical trials^16–18^. Another important concept, dataset coverage, assesses whether there are a sufficient number of data points for all relevant combinations of attributes of interest^15,19,20^. Because the notion of “sufficient” depends on the downstream uses of data and our aim is to build a desirable cohort independent of specific downstream uses, we adapt the definition of coverage. Thus, we define coverage as the number of data points in the smallest group. The uniform distribution maximizes coverage because it contains equal proportions of all groups, so we measure coverage as the similarity between a cohort’s distribution of attributes and the uniform distribution over the same attributes. The uniform distribution also maximizes entropy, which has been used to assess coverage and improve algorithmic fairness^21,22^. Moreover, the uniform distribution may be considered a baseline target by which to assess representation when a target population is not known, as each group makes up an equal proportion of the dataset.

We formulate representation and coverage as similarities to distinct target distributions. Thus, we may measure both representation and coverage using the same technique. This similarity yields results that may be compared with each other and offer desirable properties for dual objective optimization. We use Kullback-Leibler divergence (KLD) to measure the similarity of a recruited cohort to either the U.S. Census population (when assessing representation) or the uniform distribution (when assessing coverage). KLD is easily related to distributional entropy and does not require distance to be defined within the probability space, in contrast to earth mover’s distance. In an illustrative example, let a target population (*Q* in KLD(*P*∣∣*Q*)) be 40% ‘Young’ and 60% ‘Old’. A cohort (*P* in KLD(*P*∣∣*Q*)) that is 40% ‘Young’ and 60% ‘Old’ is optimally *representative*, with KLD of 0 to the target and KLD of 0.02 to the uniform. Likewise, a cohort that is 50% ‘Young’ and 50% ‘Old’ is optimally *covering*, with KLD of 0.02 to the target and KLD of 0 to the uniform. Finally, a cohort that is 45% ‘Young’ and 55% ‘Old’ would be somewhere in between, with KLD of 0.005 to both the target and uniform. Thus, KLD enables a unified framework for assessing representation and coverage. Having a unified framework promotes concrete recruitment goals, which can improve recruitment of UBR groups^23^. As stated in their core values, the *All of Us* Research Program aims to “reflect the rich diversity of the United States”, specifically including UBR groups^24^. To this end, *All of Us* has set recruitment targets of 45% of participants from racial and ethnic minorities and 75% from UBR groups more generally^25,26^. Notably, *All of Us* does not claim to be perfectly representative of the U.S. population. For comparison, a perfectly Census-representative cohort would have 37.28% of participants identifying as a racial and/or ethnic minority and 54.63% from UBR groups as as determined by race, ethnicity, and age. A perfectly covering cohort, which is more of a mathematical construct than a realistic recruitment goal for the vast majority of studies, would have 90% of participants from racial/ethnic minorities and 93% from underrepresented groups. Thus, *All of Us*’s recruitment goals aim to strike a balance between representativeness and coverage.

Several recent efforts have been made to improve representation and coverage, with specific emphasis on large biomedical data repositories. Strategies such as training patient navigators can help address barriers to participation^27,28^ in biomedical research studies. Involving participants in the design and execution of research projects has been shown to improve representation, coverage, study design, and communication between researchers and participants^29–31^. Conducting recruitment at multiple types of locations (e.g., academic hospitals and community centers) can also improve representativeness^32,33^. *All of Us* employs many of these efforts, as well as participant-centric enrollment processes, at scale^25,34^. Computational methods have been proposed to improve representation and coverage in sampling or recruitment. Nargesian et al. develop a method for achieving a target data distribution from multiple sources but may require discarding data, which may be undesirable (due to costs) in a study that recruits participants^35^. Selecting a representative cohort under competing objectives has also been explored: Flaginan et al. propose a method for selecting citizen’s assemblies that balances representativeness with equality of selection probability for individual members^36^. However, prospective recruitment fundamentally differs from selection, and the expected improvements in representativeness may differ by an order of magnitude or more^37,38^. The outside work most similar to ours is by Theodorou et al.^22^, who prioritize clinical trial recruitment sites through a reinforcement learning model which uses a reward function encoding enrollment numbers and participant diversity. Their definition of participant diversity is entropy, which is directly and negatively proportional to our formulation of coverage as KLD to the uniform distribution. However, Theodorou et al. assess diversity only by race and do not optimize for population representativeness. Representativeness, alongside coverage, may be of interest for large-scale biomedical datasets like *All of Us* because the distribution of attributes within the cohort should approximate that of the general population. Our recruitment model also assumes that recruitment resources can be re-allocated to different sites, so we do not optimize for enrollment numbers.

We situate this work to build upon the successes of *All of Us*. Although there are numerous participatory biomedical datasets and initiatives^39–41^, *All of Us* stands out due to its nationwide reach, broad range of participants, and its commitment to representing all Americans^42,43^. As a result, *All of Us* successfully includes most UBR groups near or above their Census proportions. In a study at one *All of Us* site, individuals from UBR groups had a higher chance of joining the program when approached for recruitment than their overrepresented counterparts^44^. This inclusion means that *All of Us* is substantially more covering and representative than many similarly sized and scoped biomedical datasets^26^. What remains unknown – and is the focus of this paper – is whether *All of Us*’s final cohort was as representative and covering as it could have been, given the recruitment sites available and their demographics over the course of the program. In a sense, this counterfactual question is unknowable: had the program pursued a different recruitment strategy, we do not know the precise impact that strategy would have had on the final cohort. Yet, through simulation, we approximate an answer to this question. We study how a hypothetical *All of Us* cohort would have looked if recruitment resources could be reallocated freely between sites. We also study the inherent competition between Census representativeness and coverage and discuss how to balance these two goals.

Through a case study of *All of Us*, we show how computational modeling could guide recruitment and further improve cohort representativeness and coverage. We treat participant recruitment, namely the efforts of recruitment staff, as a resource budget that may be distributed across recruitment sites. By strategically allocating recruitment resources over time, a study team may be able to recruit a more representative and covering cohort than a static or non-informed approach. In our prior work, we have shown this approach to be more effective than random or heuristic resource allocation methods, in a simplified setting, at recruiting simulated cohorts from a network of medical centers^38^.

However, those simulations distributed resources among only nine sites, compared to fifty in *All of Us*, which likely will make it more challenging for the algorithm to find the optimal solution in *All of Us*. That study also could not assess the distribution of participants who were willing to join a study (i.e., the response distribution), which may differ from the overall patient demographics at a medical center and is directly measurable in *All of Us*. Most saliently, the recruitment process in our prior study was purely hypothetical, meaning there was no real recruitment baseline to compare against. In this study, we use the actual recruitments of *All of Us* as its own baseline to demonstrate the potential further benefits of adaptive recruitment resource allocation.

To summarize, this study makes two primary contributions: first, we operationalize the goals of coverage and representation as a dual-objective optimization; second, we demonstrate, through simulation, how this optimization could be used to adapt recruitment strategies and achieve these goals. Using historical participant recruitment data from *All of Us*, we present counterfactual simulations of how the cohort could have been recruited using our strategic recruitment methodology. Moreover, we assess how our methodology aligns with *All of Us*’s stated recruitment goals to improve representation from UBR groups. Finally, we show how our recruitment methodology is robust to varied simulation parameters, representing a wide range of realistic scenarios.

## Results

### Current state of *All of Us*

Throughout our results, we focus on the subset of *All of Us* participants who have a defined 3-digit electronic health record (EHR) site code, obtained from variable src_id^45^. This subset of participants originates from EHR recruiting sites, so it is the subset that is theoretically modifiable through resource allocation. We find that this subset of participants is qualitatively similar to the overall *All of Us* cohort (Table 1 and Supplementary Table 3). This constraint is relaxed to include participants without a defined EHR site in Supplementary Section 1.

**Table 1:** Participant demographics in *All of Us* compared to the U.S. Census

Demographic Group	*All of Us* Count (%)	Census Count (%)
Age		
20-44	81,001 (30.02)	109,958,279 (44.27)
45-64	98,520 (36.51)	81,287,170 (32.73)
65+	90,341 (33.48)	57,115,071 (23.00)
Gender		
Female	165,970 (61.50)	126,509,045 (50.94)
Male	103,892 (38.50)	121,851,475 (49.06)
Race		
Asian	10,203 (3.78)	16,155,290 (6.50)
Black	64,330 (23.84)	32,828,357 (13.22)
Native Hawaiian / Pacific Islander	630 (0.23)	607,035 (0.24)
Two or More Races	9,384 (3.48)	5,312,304 (2.14)
White	185,315 (68.67)	193,457,534 (77.89)
Ethnicity		
Hispanic / Latino	53,292 (19.75)	41,378,366 (16.66)
Non-Hispanic / Latino	216,570 (80.25)	206,982,154 (83.34)

We restrict our cohort to participants who have a 3-digit EHR site code defined for at least one of their observations.

**Fig. 1:**
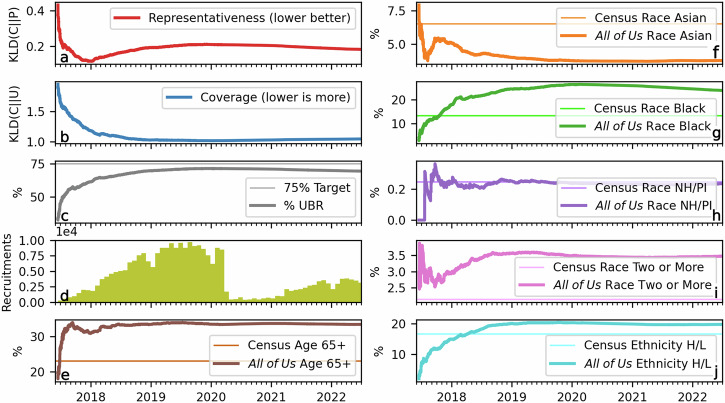
Temporal trends in the representativeness, coverage, group proportions, and participant counts for the *All of Us* cohort with defined EHR sites. In panels (**a**, **b**), we show the Kullback-Leibler divergence to the Census (KLD(*C*∣∣*P*)) and uniform (KLD(*C*∣∣*U*)) distributions, highlighting our measures of representativeness and coverage, respectively. Panel (**c**) tracks the progress of *All of Us* towards its goal of 75% participants from underrepresented in biomedical research (UBR) groups (of attributes we can measure) while panel (**d**) shows the number of recruited participants over time. Panels (**e**–**j**) detail the proportions of the UBR groups which we can measure in this analysis, along with each group’s Census proportion. NH/PI refers to Native Hawaiian/Pacific Islander race while H/L refers to Hispanic/Latino ethnicity.

We begin our case study into *All of Us* recruitment by approximating the program’s goals through its recruitment patterns. Throughout the results, *C* refers to a cohort’s demographic distribution by age, gender, race and ethnicity, while *P* refers to the Census distribution over those attributes and *U* refers to the uniform distribution. KLD(*C*∣∣*P*), the Kullback-Leibler divergence from the cohort distribution to the Census, assess representativeness while KLD(*C*∣∣*U*), the divergence from the cohort distribution to the uniform, assess coverage. Lower divergence values are considered to be more representative and covering, respectively. In the first seven months (up to January 2018) of recruitment, *All of Us* substantially improved representativeness, coverage, and recruitment of UBR populations (Fig. 1a, b). These improvements may be attributed to almost all UBR subgroups (by age, race, or ethnicity) being recruited at levels matching or exceeding their Census levels of representation. The following two years of recruitment (January 2018–2020) continue this prioritization of UBR groups, especially for Black and Hispanic/Latino participants. As expected, this recruitment strategy improved cohort coverage at the cost of Census representativeness. Presumably due to the COVID-19 pandemic, recruitment substantially slowed from March 2020–2021, and then resumed from April 2021 onward.

Interestingly, the post-pandemic recruitment strategy appeared to reverse course. The proportion of Black and Hispanic/Latino participants decreased somewhat from respective 2020 maxima of 26.40% and 20.37% to respective final cohort percentages of 23.84% and 19.75%. Concomitantly, coverage (KLD(*C*∣∣*U*)) and proportion of UBR groups decreased while Census-representativeness improved (Fig. 1a–c, g, j). Another notable result is that the proportion of Asian participants appeared to follow an opposite trend compared to other UBR races and ethnicities. Except for an early uptick in late 2017, the proportion of Asian participants in the *All of Us* cohort monotonically decreased throughout most of the recruitment process, with the group’s final representation substantially below its Census level. This decreasing representation of Asian participants in the *All of Us* cohort highlights an opportunity the program may wish to consider. Sites with substantial underrepresentation of Asian participants contribute more cumulative participants to *All of Us* over time (Supplementary Section 2), so counteracting this effect through strategic recruitment may improve both representation and coverage.

The demographics of the final *All of Us* cohort generally align with the program’s goals. In *All of Us*’s total final cohort, 66.58% of the participants come from groups who are UBR as determined by age, race, or ethnicity, including 43.61% from racial and/or ethnic minorities. When we limit the dataset to participants with a defined EHR site, like we do for most of our analyses, the proportion of participants from underrepresented groups increases to 69.42% total and 45.15% from racial/ethnic minorities. These proportions approach or exceed *All of Us*’s recruitment goals of 75% UBR participants and 45% from racial/ethnic minorities. Notably, some attributes that define UBR groups in *All of Us*’s definition (e.g., income, sexual orientation, or access to healthcare) are not studied in this analysis because they are not available in the Census dataset studied. Moreover, participants who self-identify as a race that aligns with the Census category of American Indian or Alaska Native are not included in this version of *All of Us* data.

**Fig. 2:**
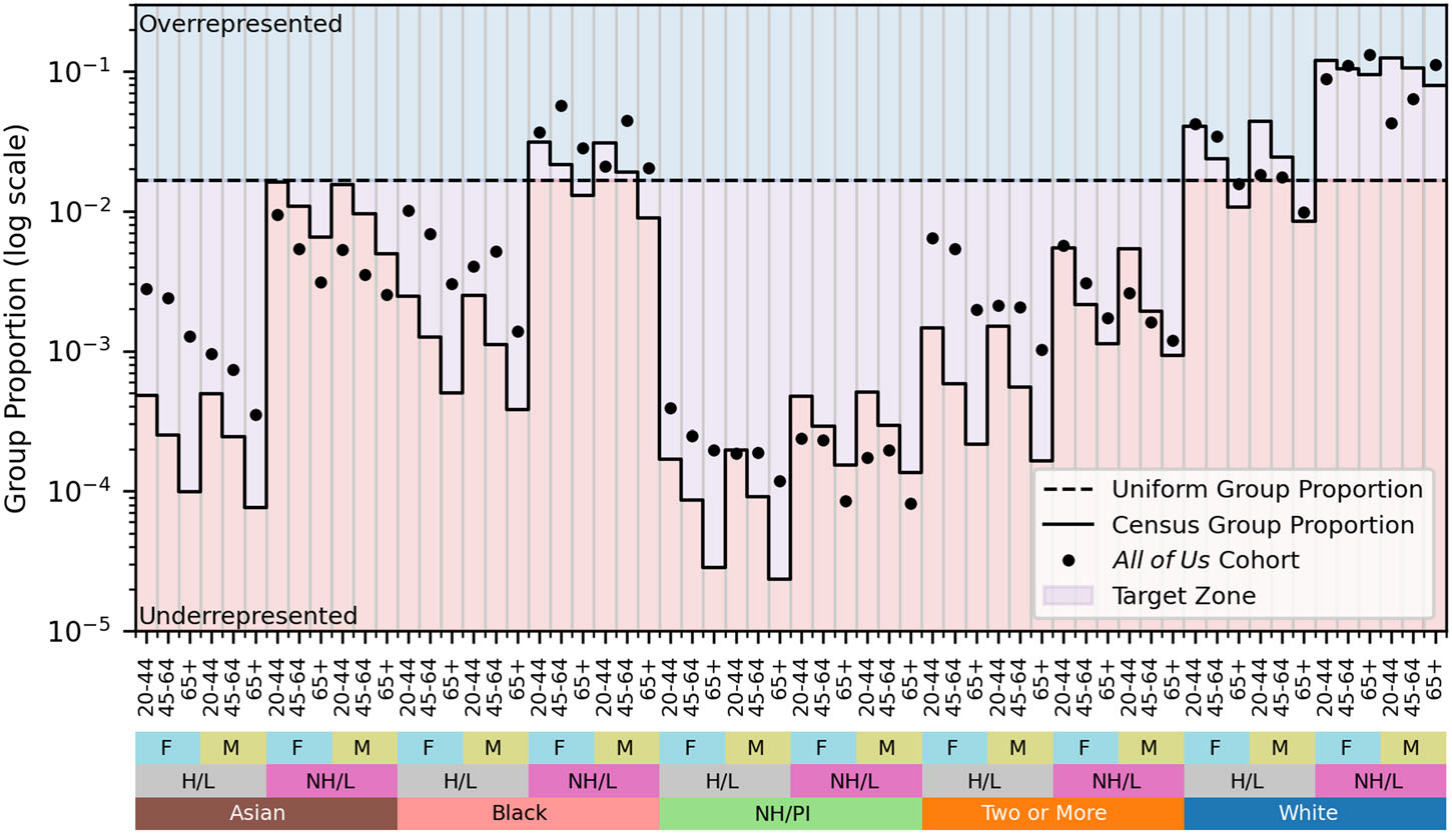
*All of Us* participant demographics for participants with a defined EHR site. Absolute proportions of each combined demographic group are shown as black dots and compared to a uniform distribution (black dashed line) and each group’s proportion in the U.S. Census (black solid line). Target group proportions are between the uniform and Census proportions (purple shaded area), while proportions above both lines are considered overrepresented and proportions below both lines are underrepresented. F refers to female, M refers to male, (N)H/L refers to (non-)Hispanic/Latino, and NH/PI refers to Native Hawaiian / Pacific Islander.

The demographics of the final *All of Us* cohort align with our formulated goals of representativeness and coverage. Because our combined objective function in Equation (5) balances representation and coverage, the ideal cohort proportion for each demographic group will lie somewhere between its Census and uniform proportions. Thus, we compare the proportion of each fully defined demographic group (e.g., 20–44 year old Asian Hispanic/Latino women) in the *All of Us* cohort to that group’s proportion in the Census and to the uniform distribution (Fig. 2). Of the 60 combined demographic groups studied, 35 (58%) were represented somewhere between their Census and uniform levels, and several more were only slightly outside these bounds. This subgroup representation appears to indicate that *All of Us* prioritized representativeness and coverage as we have defined it. Certain UBR groups (e.g., Black, Hispanic/Latino, or older participants) are intentionally overrepresented above their Census proportions in this cohort (Table 1 and Fig. 2). However, a few demographic groups remain consistently underrepresented in the *All of Us* cohort, particularly non-Hispanic/Latino Asian or Native Hawaiian / Pacific Islander participants. Younger and male participants also tended to be less represented in the cohort compared to older and female participants. Underrepresentation compounds across demographic factors: within the underrepresented Non-Hispanic/Latino Asian and Native Hawaiian / Pacific Islander subgroups, men are consistently represented less than women. Strategic recruitment strategies may also improve the representation of these groups, in addition to the hypothesized improvement in the representation of Asian participants discussed earlier.

### Understanding the recruitment sites available

To propose strategies for improving *All of Us* cohort representativeness and coverage, we must first understand the recruitment sites available, which we term the policy space. As detailed in Section “Defining recruitment sites and prior knowledge”, we identify 50 EHR sites to constitute our policy space. Each site has its own response distribution that captures the demographics of participants potentially willing to join *All of Us*. We generate these response distributions using historical recruitment data from each site in *All of Us*. Although site response distributions varied somewhat over time, the vast majority of this variation is random. Only 0.233% of site demographic combinations had a statistically significant (*α* = 0.05, multiple hypothesis correction not supported) trend over time when assessed with a Kwiatkowski-Philips-Schmidt-Shin (KPSS) test with null hypothesis of stationary data around a constant^46^. Thus, we utilize each site’s cumulative historical recruitments up to the simulated date (e.g., recruitment data through January 2019 if the simulated recruitment step is in January 2019) to mitigate random distribution variation over time.

**Fig. 3:**
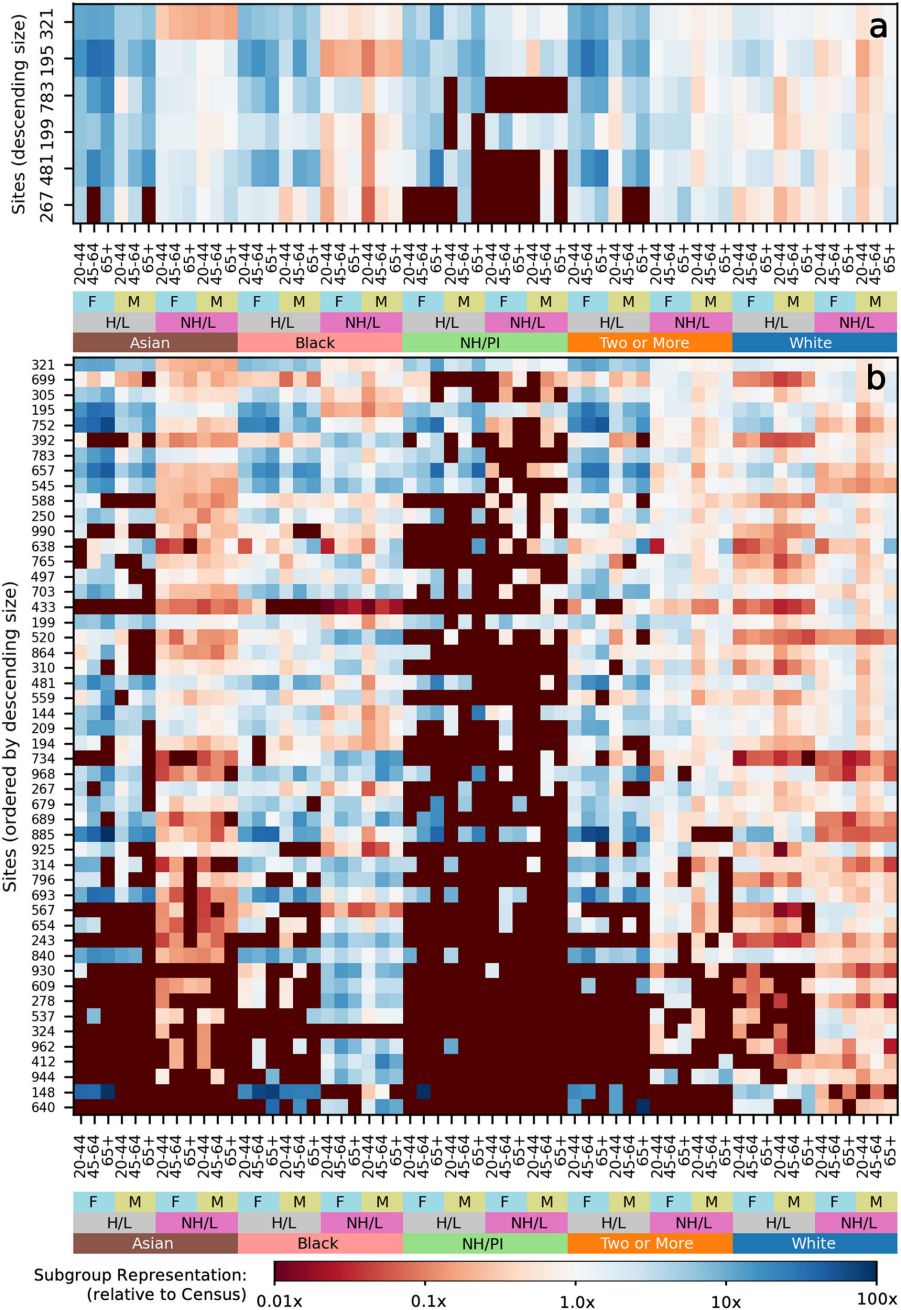
Final response distributions for EHR sites relative to U.S. Census values. Six selected sites are shown in (**a**) while all 50 EHR sites are shown in (**b**). Each row corresponds to one site, sorted by descending number of participants, while each column corresponds to a fully defined demographic group. Blue colors indicate a group is overrepresented (relative to its Census proportion) at a site while red colors indicate a group is underrepresented. The deepest red color indicates there are no participants from that demographic group at that site. F refers to female, M refers to male, (N)H/L refers to (non-)Hispanic/Latino, and NH/PI refers to Native Hawaiian / Pacific Islander.

**Fig. 4:**
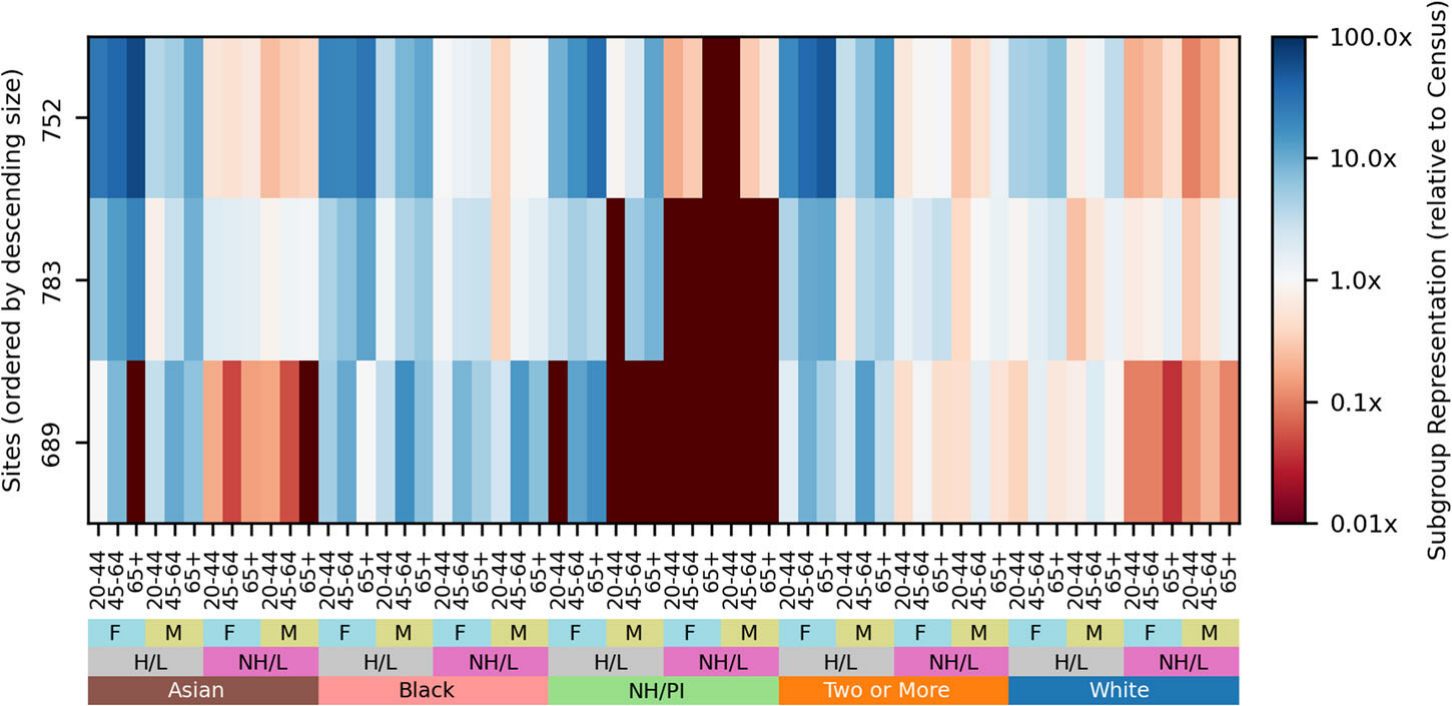
Differences in response distributions for the three EHR sites in New York, NY. Blue colors indicate a group is overrepresented (relative to its Census proportion) at a site while red colors indicate a group is underrepresented. The deepest red color indicates there are no participants from that demographic group at that site. F refers to female, M refers to male, (N)H/L refers to (non-)Hispanic/Latino, and NH/PI refers to Native Hawaiian / Pacific Islander.

Figure 3 shows the final response distributions at each site, relative to the U.S. Census proportions of each demographic group. Few sites represent non-Hispanic/Latino Asian or Native Hawaiian / Pacific Islander participants at or above their Census levels, and several sites lack Native Hawaiian / Pacific Islander participants entirely. The Census proportions of these demographic groups are also far below the uniform distribution, so their underrepresentation would negatively impact both Census representativeness and coverage. Some sites differ from these overall trends. Sites 783, 195, 481, and 267 all represent non-Hispanic/Latino Asian participants near or above their Census levels, while sites 321, 195, and 199 have substantially better representation of Native Hawaiian / Pacific Islander participants than other sites (Fig. 3a). Thus, trends of underrepresentation may potentially be overcome by strategically prioritizing recruitment at certain sites. In Table 2, we summarize the geographical information about each EHR site, including the most common 3-digit ZIP codes (ZIP3s) of participants from the site and the major cities in the region. Eleven major cities have multiple EHR sites: Atlanta, GA (2); Birmingham, AL (2); Boston, MA (2); Chicago, IL (7); Jackson, MS (2), Los Angeles, CA (2); Milwaukee, WI (2); New Orleans, LA (2); New York, NY (3); Phoenix, AZ (2); and San Diego, CA (2). Even within the same geographic area, recruitment sites may have substantially different response distributions. New York City’s three sites have notable differences: site 752 is the only site which represents Native Hawaiian / Pacific Islander participants at all while site 783 is the only site to represent Asian participants at or above their Census levels (Fig. 4). Other cities with multiple EHR sites also show differences in their participant response distributions.

**Table 2:** The most common geographic regions of home addresses for participants from each *All of Us* EHR site, defined by the first 3 digits of ZIP code (ZIP3) as well as corresponding major cities^57^

Site	Most Common ZIP3(s)	City or Cities
144	919–922, 925	San Diego, CA
148	532	Milwaukee, WI
194	010–017	Springfield, MA and Worcester, MA
195	906, 925–928	Riverside, CA and Santa Ana, CA
199	952, 956–959	Sacramento, CA
209	900–905, 913–914	Los Angeles, CA
243	390–397	Jackson, MS
250	750–753, 765–767	Dallas, TX; Waco, TX
267	940–949	San Francisco, CA
278	367	Selma, AL
305	017–024	Boston, MA
310	300–307	Atlanta, GA
314	290–299	Columbia, SC
321	850–853, 857	Phoenix, AZ and Tucson, AZ
324	546, 669	La Crosse, WI and Rochester, MN
392	350–355, 359	Birmingham, AL
412	700–706	New Orleans, LA
433	544–549	Wausau, WI
481	900–902, 910–911	Los Angeles, CA and Pasadena, CA
497	600–606	Chicago, IL
520	601–608	Chicago, IL
537	354	Tuscaloosa, AL
545	601–608	Chicago, IL
559	320–326, 344	Jacksonville, FL and Gainesville, FL
567	548, 557–558	Duluth, MN
588	493–495	Grand Rapids, MI
609	356–358	Huntsville, AL
638	551–554, 640–641, 660–661, 850	Minneapolis, MN, Kansas City, MO, and Phoenix, AZ
640	606	Chicago, IL
654	376–379	Knoxville, TN
657	330–334	Miami, FL
679	601–606	Chicago, IL
689	100, 104, 112–114	New York, NY
693	060-069	New Haven, CT
699	150–156, 166–168	Pittsburgh, PA
703	019–023	Boston, MA
734	300–303	Atlanta, GA
752	100, 104–108, 112	New York, NY
765	480–485	Detroit, MI
783	100, 110–113	New York, NY
796	700–704	New Orleans, LA
840	105–109, 124–127	White Plains, NY
864	530–534	Milwaukee, WI
885	919–921	San Diego, CA
925	600–607	Chicago, IL
930	390–396	Jackson, MS
944	191	Philadelphia, PA
962	350–352	Birmingham, AL
968	601–608	Chicago, IL
990	537–539, 610–611	Madison, WI and Rockford, IL

### Identifying a representation and coverage goal

**Fig. 5:**
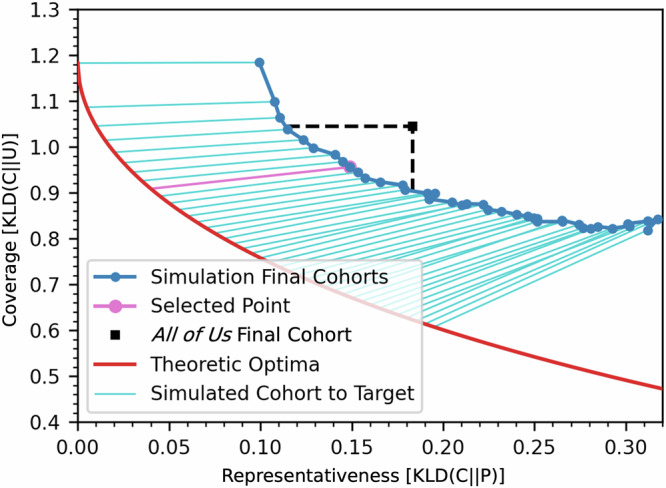
Simulated final cohorts (dark blue dots and line) with EHR-site-based policy for target points on the representativeness-coverage pareto optimal frontier (red line). The *All of Us* final cohort is shown as a black dot, with black dashed lines extending its KLD(*C*∣∣*P*) and KLD(*C*∣∣*U*) values outwards. Cyan lines show the relationship between a cohort’s final representativeness, coverage, and target point. The pink line and pink-outlined point indicate the final selected target point of (0.04, 0.909).

We operationalize the goals of cohort representativeness and coverage as a dual objective optimization detailed in Section “Balancing representation and coverage”. As a result, there are infinitely many optimal combinations of representativeness and coverage that arise from differentially weighting these two objectives. Thus, two optimal cohorts may have starkly different demographic distributions, and identifying a specific recruitment goal is critical. We specify a recruitment goal that balances representativeness and coverage such that both measures may be improved relative to the historical final *All of Us* recruited cohort. By doing so, our recruitment goal aligns with the apparent balance of representativeness and coverage that *All of Us* desires; it does not represent a major shift towards prioritizing either Census-representativeness or coverage. To empirically identify this recruitment goal, we run our recruitment algorithm with 40 different target combinations of representativeness and coverage. Under the constraints of our simulation, the final cohorts that could be recruited by strategically allocating resources to EHR-based sites are more representative and/or covering than the historic *All of Us* cohort, but they do not reach the theoretic Pareto frontier (Fig. 5). These results are expected because of the practical limitations of recruiting from a finite number of sites with unique response distributions. Eleven of our 40 simulated final cohorts exceed both the representativeness and coverage of the *All of Us* historical cohort, shown in the area below and to the right of the dashed black lines in Fig. 5. We empirically select the sixth (of eleven) target point in this region, corresponding to ${\rm{KLD}}\left(\right.C\,| | \,P{\left)\right.}_{T}=0.04\ {\rm{and}}\ {\rm{KLD}}\left(\right.C\,| | \,U{\left)\right.}_{T}=0.909$, as the target balance of representativeness and coverage in subsequent experiments.

### Recruiting a more representative and covering cohort through strategic resource allocation

Recognizing the numerous successes of *All of Us* in recruiting UBR populations, we aimed to evaluate whether strategic resource allocation could further improve the representativeness and coverage of *All of Us*. We present a counterfactual simulation of how recruitment could have unfolded from the inception of *All of Us* until June 2022; the same total number of participants are recruited in simulation, but their recruitment sites – and, thus, demographics – differ. In our simulation, we assume that *All of Us* has the ability to update their recruitment policy on a quarterly basis, for a total of 21 3-month recruitment iterations from 2017 to 2022. We repeat each simulation 40 times and report the mean and 95% Bayesian credible interval (CI) for all results of interest.

**Fig. 6:**
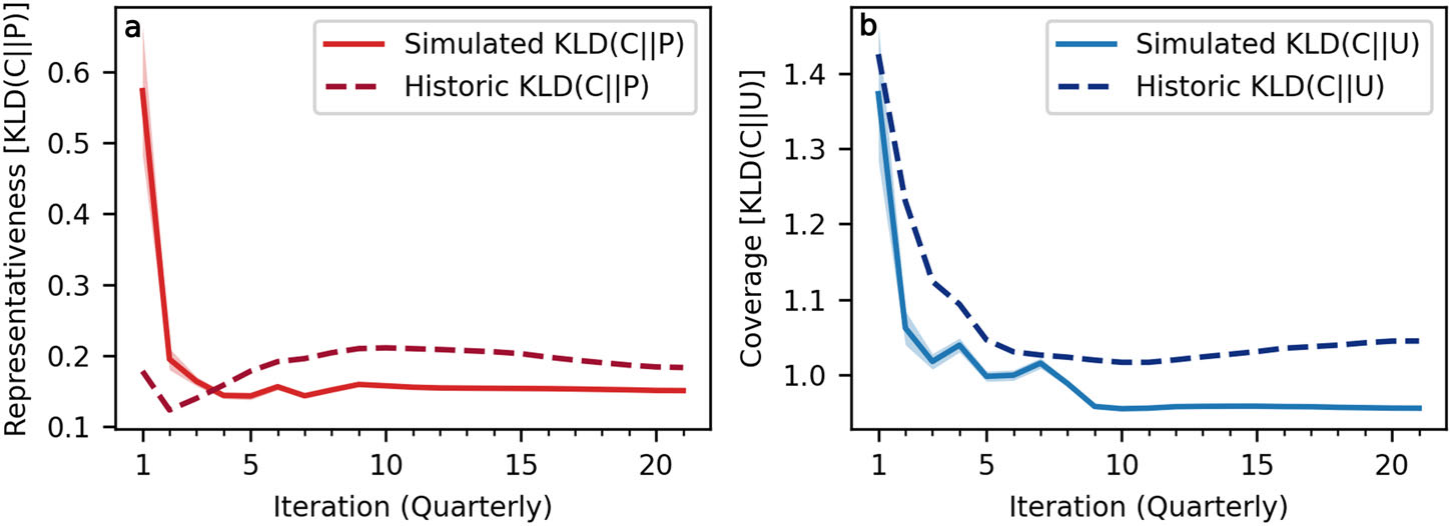
Strategic recruitment resource allocation improves both representativeness and coverage. Representativeness (KLD(*C*∣∣*P*), (**a**) and coverage (KLD(*C*∣∣*U*), (**b**) for simulated *All of Us* cohorts are compared to historical recruitment. The solid red line depicts simulated cohort representativeness over time with the shaded region indicating the 95% Bayesian credible interval while the dashed red line depicts *All of Us*'s historical representativeness. The solid blue line depicts simulated cohort coverage over time with the shaded region indicating the 95% Bayesian credible interval while the dashed blue line depicts *All of Us*'s historical coverage.

Despite starting without any knowledge of site response distributions, our model improves the coverage of *All of Us* as early as the first quarter of recruitment and improves upon representativeness after the fourth quarter (Fig. 6). These improvements are maintained throughout the recruitment process, producing a final cohort that is significantly more representative and covering than *All of Us*’s final cohort with mean final simulation KLD(*C*∣∣*P*) = 0.1508 [95% CI 0.1496–0.1520] and KLD(*C*∣∣*U*) = 0.9557 [95% CI 0.9550–0.9564] compared to *All of Us* KLD(*C*∣∣*P*) = 0.1834 and KLD(*C*∣∣*U*) = 1.0450. Two-sided Wilcoxon signed-rank tests comparing the mean final cohort representativeness and coverage to the null hypothesis of the *All of Us* baseline yield *M* = −20 (all 40 replicates less than the null hypothesis) and p-values of 1.819 × 10^−12^ for each measure.

**Fig. 7:**
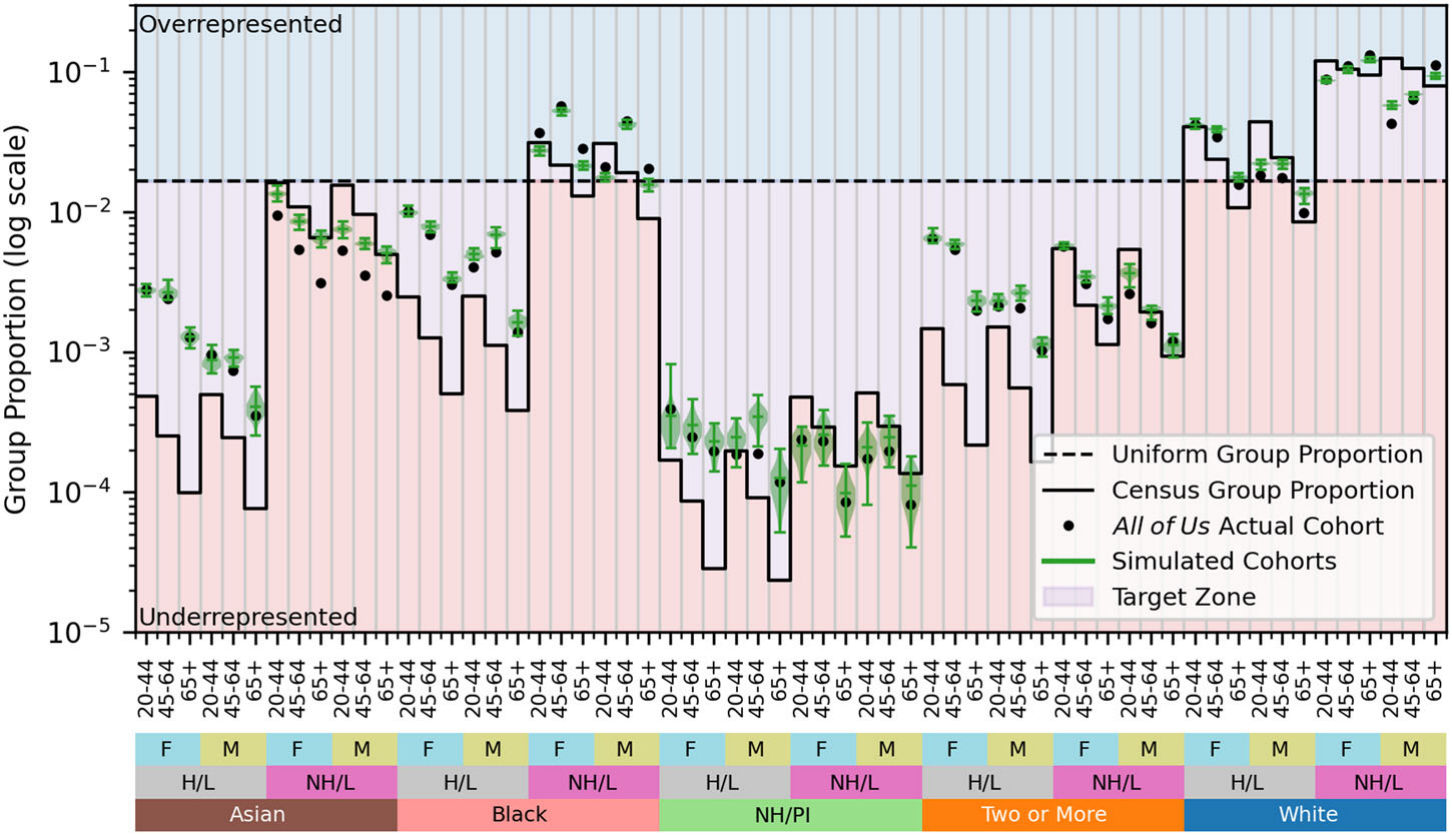
Strategic recruitment resource allocation markedly improves subgroup-specific representation in simulated cohorts compared to the *All of Us* historical cohort. Demographic groups in the final *All of Us* historical cohort are represented by black circles while groups in the mean simulated final cohort for strategic recruitment are depicted with green violin plots. The caps of each violin plot indicate the maximum and minimum proportion of a group across the 40 experimental replicates, while the middle line indicates the group’s mean proportion and the shaded region shows the distribution of the group’s proportion across experimental replicates. A subgroup is considered to be within target levels of representativeness and coverage if its proportion is between its Census and uniform levels. F refers to female, M refers to male, (N)H/L refers to (non-)Hispanic/Latino, and NH/PI refers to Native Hawaiian / Pacific Islander.

We further confirm these results by studying subgroup-specific proportions in the final mean simulated cohort compared to the actual *All of Us* cohort. Although *All of Us* historical recruitment produced a cohort in which 35 of 60 (58%) demographic groups were represented between their Census and uniform levels (i.e., the target zone), our strategic allocation of resources produced a cohort in which 41 of 60 (68%) groups had proportions in the target zone (Fig. 7). Even when strategic recruitment could not push a group into target zone levels of representation, the simulated cohort was typically closer to the target zone than the historical final *All of Us* cohort. This effect is particularly noticeable within the non-Hispanic/Latino Asian, Native Hawaiian / Pacific Islander, and multiracial subgroups, who are more represented under strategic recruitment.

As an additional measure of representation, we study the proportions of our simulated cohorts that are considered underrepresented in biomedical research, and the subsets which are underrepresented by race and ethnicity. These are measures that *All of Us* has used for its recruitment goal setting, aiming to have 75% of the cohort UBR and 45% underrepresented by race or ethnicity. We find our simulated cohort to, on average, include 68.20% [95% CI 68.10%–68.31%] UBR populations, of which 46.71% [95% CI 46.65%–46.77%] are racial and/or ethnic minorities. Although the total proportion of UBR populations is slightly lower than the historical *All of Us* final cohort (at 69.42%), the simulated cohort has a higher proportion of participants identifying with racial/ethnic minorities than *All of Us* (at 45.15%). Given that our assessment of underrepresentation is limited to age, race, and ethnicity, the total percentage of participants from underrepresented groups in our final simulated cohort would likely be higher.

**Fig. 8:**
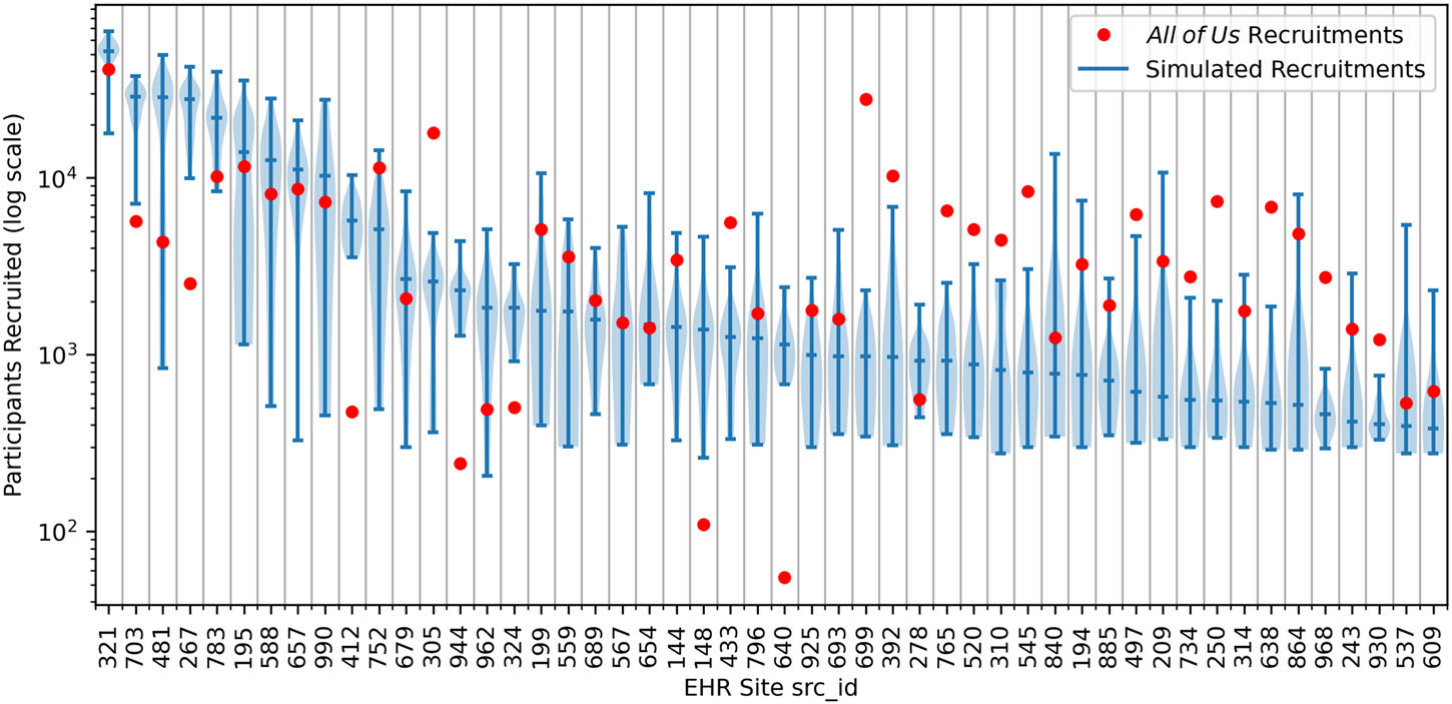
Recruitment resources (i.e., participant equivalents) allocated to each EHR site in simulation. Blue violin plots show the distribution of simulated resources allocated while historical allotments in *All of Us* recruitment are denoted by red dots. The caps on each blue line indicate the minimum and maximum recruitments at that site across 40 simulations, while the middle cross-line indicates the median recruitment number, and the shaded region indicates the distribution of recruitments at each site.

To study how our model for strategic recruitment improves cohort representativeness and coverage, we analyze how it allocates resources among available recruitment sites. In particular, we study the EHR sites (each src_id) that are prioritized or de-prioritized for recruitment compared to *All of Us*’s historical baseline. We show a violin plot of each site’s recruitment resource allocation (measured in number of participants recruited) in simulation and compare these allocations to the historical number of participants recruited from each site in *All of Us* (Fig. 8). Patterns of resource allocation to certain sites vary greatly across experimental replicates. For instance, the resources allocated to site 195 are roughly uniformly distributed from recruitment counts of 1000 to almost 30,000. This likely reflects the adaptive response of our recruitment strategy to stochasticity in recruitment simulation, prioritizing or de-prioritizing sites as needed.

Overall, most sites have similar recruitment numbers in our simulation as they did historically in *All of Us*: 29 of 50 sites (58%) have an experimental resource allocation range that encompasses the site’s historical recruitment number (Fig. 8). Yet, the 42% of sites that would receive substantially different amounts of resources highlight where adaptive recruitment can improve representativeness and coverage. Some sites (e.g., 703, 267, 412, and 944) are recruited from substantially more often in simulation than they were historically. By referencing Fig. 3, we can gain insight into this prioritization. Sites 703, 412, and 944 all represent non-Hispanic/Latino Black participants at a greater rate than the Census, which addresses a limitation of the most highly prioritized site (both historically and in simulation), 321. Likewise, site 267 has some of the highest representation of non-Hispanic/Latino participants identifying as Asian or with multiple races, who are not as highly represented at other sites. Other sites (e.g., 699, 305, 250, and 638) are recruited from substantially less often in simulation than historically. While site 699 generates the second highest number of recruitments in *All of Us* historically, its response distribution has relatively low levels of UBR groups. Sites 305 and 250 generally follow similar demographic trends to the highly-prioritized site 321 but lack some of site 321’s unique benefits like high representation of Native Hawaiian / Pacific Islander participants. Finally, site 638 shows uniquely pronounced differences in representation by age, with older individuals being more highly represented at the site across all race, ethnicity, and gender groups. While this may align with *All of Us*’s goals to recruit individuals aged 65 and up, older adults are generally well-represented at other sites, so the benefit of the site is somewhat blunted.

Interestingly, some geographic regions have at least one site that is prioritized while another is de-prioritized, compared to the historical *All of Us* baseline. For instance, site 703 is prioritized while site 305 is de-prioritized, despite both sites covering similar ZIP3s in the Boston, MA area. The distributions at these two sites are broadly similar, with one exception: site 703 has a greater proportion of non-Hispanic/Latino Black participants (relative to the Census) while site 305 has a lower proportion. Relatively limited differences in response distributions have magnified impact on cohort representativeness and coverage, and our recruitment strategy is able to identify and leverage these differences.

### Varying simulation parameters

For clarity and brevity, we presented results for one policy space and set of simulation parameters that balanced realism with experimental simplicity. To validate that our results hold under a variety of recruitment conditions, we systematically modified several key simulation components. We tabulate some summary results of these analyses in Supplementary Table 1 and provide further cohort details in the Supplementary Information. Briefly, including participants who do not have a defined EHR site modifies final cohort characteristics but does not invalidate improvements in representation and coverage from adaptive recruitment. Constraining the model to be less flexible in resource allocation or only allowing annual (instead of quarterly) policy updates hampers, but does not eliminate, the model’s adaptive recruitment ability. Modifying the recruitment goal to only prioritize representativeness or coverage achieves the desired effect at the cost of the other objective. Models prioritizing coverage yield final cohorts with the highest proportion of UBR populations of any experiment we ran, as expected. When compared to a uniform resource allocation policy baseline, our methodology yielded significantly more representative and covering final cohorts. Lastly, we considered the stochastic effects of varying demographic imputations in Supplementary Table 2, which had little discernible impact on results.

## Discussion

Significant efforts and funding have been employed to ensure biomedical research adequately generalizes to everyone. One facet of these efforts has been improving the representation of understudied (i.e., UBR) populations in biomedical datasets. *All of Us* exemplifies these efforts, aiming to “build one of the most diverse health databases in history”^24^. To this end, *All of Us* has stated recruitment goals as 75% of participants from UBR groups and 45% from racial and ethnic minorities^25,26^. These proportion goals provide a straightforward assessment of *All of Us*’s cohort and can evaluate progress over time. However, aggregate proportion goals lack the specificity to determine *which* groups are under- or overrepresented in detail. Thus, these measures have limited utility for planning recruitment strategies. More detailed statistical distance measures derived from entropy have been used to assess cohort representativeness to a target population and guide recruitment efforts in simulation^22,38,47^. In this paper, we extended this methodology to a dual objective problem, prioritizing both Census-representativeness and coverage. By doing so, we address a key limitation of Census-representative datasets: a perfectly Census representative cohort may only have a few individuals from particularly small groups. For example, a perfectly Census representative 1000-person cohort would only contain 18 participants aged 85 or over (U.S. Census Table S0101). Such numbers may be insufficient for statistical power or to train a fair machine learning algorithm^11^. The insufficiency of proportionate representation can further magnify when smaller groups are differentially impacted by disease^48^. When we recruited a cohort using only representativeness as the objective, the proportion of UBR individuals was substantially lower than any other experiments (Supplementary Table 1). By including coverage as a second objective, we balance out the blind spots of proportional representation.

The main finding of this work is that a different allocation of recruitment resources among *All of Us*’s recruitment sites may yield a final cohort that is more representative and covering than the program’s current composition. There are several potential reasons why we observe these improvements. A straightforward reason might be that *All of Us* did not allocate recruitment resources to optimize what we defined as representativeness and coverage. While we believe this reason is partly responsible for our observed improvements in simulation, other factors also play a role. It is possible and likely that *All of Us* used a different objective function for determining where to prioritize recruitment or open new sites. Because we do not know this objective function, we proposed the goals of representativeness and coverage and mathematically measured these through Kullback-Leibler divergence.

Even if we perfectly operationalized *All of Us*’s recruitment goals, our observed improvements in simulation may not manifest in reality. Our simulation necessarily simplifies real-world factors that affect recruitment, which could translate to a simulation-reality gap. For instance, 3- or 12-month cycles may be too short for resource re-allocation in practice and not reflect the realities of recruitment site staffing. To prevent massive disparities in resource allocation between sites, we constrain the ratio of maximum and minimum resource allocation to sites. Tightening this constraint has a noticeable impact on cohort representativeness and coverage (Supplementary Table 1). We also consider recruitments to be interchangeable between sites, which may not reflect differences in recruitable populations at different EHR sites. A site in a sparsely populated region may not support as many recruitments as one in a major urban center. Despite these caveats, the recruitment strategies empirically identified by our model often align with each site’s historical resource allocation. Experimentally-derived resource allocations overlap with sites’ historical recruitment counts in more than half of the EHR recruitment sites, including seven of the ten sites most heavily prioritized by our methodology. Nevertheless, sites such as 412, 148, and 640 would need to recruit about an order of magnitude more participants in simulation than reality, which may not be feasible. Even if such recruitment volume were feasible, it may change the response distribution at the site. This effect would be particularly pronounced at recruitment sites employing highly specialized recruitment strategies that may not scale easily. Likely, the improvements we observe over historical *All of Us* representativeness and coverage can be attributed to a combination of all three of these factors: a more optimized experimental methodology, a slightly different objective function, and a somewhat optimistic simulation of real-world recruitment.

In addition to the differences between our simulations and *All of Us* noted above, our work has some dataset limitations. Our assessments of representativeness and coverage are limited by data availability and harmonization between *All of Us* and the U.S. Census (Section “Datasets and harmonization”). This leads to imperfect matches like “sex” in Census data with “gender” in *All of Us* and the exclusion of individuals identifying as American Indian / Alaska Native (because they are not included in the *All of Us* dataset used) or Middle Eastern / North African (because they are not reported in Census data). We matched values as closely as possible given the dataset limitations we faced but note that these limitations reduce our ability to assess representation and coverage over these groups and lead to a consistent underestimate of UBR proportions. Likewise, a large number of participants who identify as Hispanic/Latino in both *All of Us* and the Census list “Other” (or some variation thereof) as their race. The Census Bureau imputes a race for individuals listing “Some Other Race” in the dataset we used, so we applied a similar imputation methodology to best approximate their data; however, we acknowledge that this imputation process will shift our data from the ground truth. Because our Census dataset was limited to age, sex, race, and ethnicity, other measures which define UBR groups could not be compared in this study, as discussed in Section “Current state of *All of Us”*. Thus, all total UBR proportions in this study are underestimates of the true UBR proportions. We mitigate this limitation by comparing UBR proportions within our experiments, such that the underestimation effect is consistent. A relative change in UBR proportions, then, would still be expected to yield an absolute change when a more comprehensive definition is applied.

Despite the aforementioned limitations, this work has several promising applications and future directions. Using data available to the program right now, *All of Us* can evaluate which existing sites are worth investing additional recruitment resources in to achieve their desired recruitment goals. The same methodology can also be used to determine where to open new recruitment sites as long as the program has some prior knowledge about the response demographics at the potential new site. Beyond *All of Us*, other data repositories and collectors may utilize our methodology to guide recruitment efforts for representativeness and coverage. Our methodology may be applied to multi-site studies at any scale. Another promising avenue for research could be to measure participant retention rates and adapt the recruitment strategy accordingly, which may be of particular relevance for longitudinal studies and interventional trials. Further research could analyze the impacts – expected and unforeseen – of these modified recruitment strategies. For example, studies may analyze whether more representative and covering datasets do, in fact, yield more accurate or generalizable artificial intelligence models. Other future studies may analyze the impact of strategic recruitment on data quality or define representation across additional study-relevant variables. A notable strength of our proposed methodology is that study designers may choose which variables they want to prioritize for representation and coverage, provided they have a ground truth or target value available. We opted to use broad demographic variables (age, gender, race, and ethnicity) because of their availability for comparison against Census data, but clinical or geographic variables may also be used.

In conclusion, we showed the utility of computational strategic recruitment to guide site selection and improve representation and coverage of recruited cohorts. We extended previous work in adaptive recruitment resource allocation^38^ in three notable ways: to dual-objective constrained optimization that balances representation and coverage, to actual participant response distributions using historical data from a nationwide program, and to the larger and more complex recruitment policy space in *All of Us*. With these contributions, we further bridge the gap between methodological theory and practice and provide an actionable pathway for data-collecting programs to achieve several simultaneous recruitment goals.

## Methods

All research activities described in this article were conducted in accordance with the *All of Us* Data User Code of Conduct and Data and Statistics Dissemination Policy and only authorized authors who completed *All of Us* Responsible Conduct of Research training accessed data. Per the *All of Us* Institutional Review Board, research in the Controlled Tier uses a data passport model and does not constitute research involving human subjects (Protocol 2021-02-TN-001), so IRB approval was not required for this study.

### Balancing representation and coverage

While representation and coverage are both desirable properties of a cohort, there is no cohort that can be perfectly representative of the U.S. population and perfectly covering. This impossibility stems from the fact that the U.S. population is not uniformly distributed among all demographic groups. For example, assume a cohort demographic distribution (*C*) is perfectly reflective of the demographic distribution of the U.S. population (*P*). We show that such a cohort cannot be perfectly reflective of the uniform distribution (*U*) in Equation (1), which also holds in reverse.

1 $${\rm{KLD}}(C\,| | \,P)=0\ \Rightarrow \ {\rm{KLD}}(C\,| | \,U)={\rm{KLD}}(P\,| | \,U)\,\ne \,0$$

Nevertheless, this tension between the representation and coverage terms gives way to a set of possible solutions that optimize some combination of the two objectives. Because Kullback-Leibler divergence is a convex function, we know that the Pareto frontier of our two KLD terms would admit the form of a superellipse^49^. Moreover, we know two endpoints of the superellipse must be KLD(*U*∣∣*P*) and KLD(*P*∣∣*U*), because these values each minimize one term of the dual objective function. Then, following Li et al.^49^, we empirically determine the superellipse power *n* by fitting a curve to other points on the Pareto frontier. We use the SciPy minimize function to empirically generate demographics distributions approximating combinations of KLD(*C*∣∣*P*) and KLD(*C*∣∣*U*) as shown in Equation (2).

2 $$\alpha \times \left(\right.{\rm{KLD}}\left(\right.C\,| | \,P\left)\right.\left)\right.+(1-\alpha )\times \left(\right.{\rm{KLD}}\left(\right.C\,| | \,U\left)\right.\left)\right.\ {\rm{for}}\ \alpha \in [0,1]$$

**Fig. 9:**
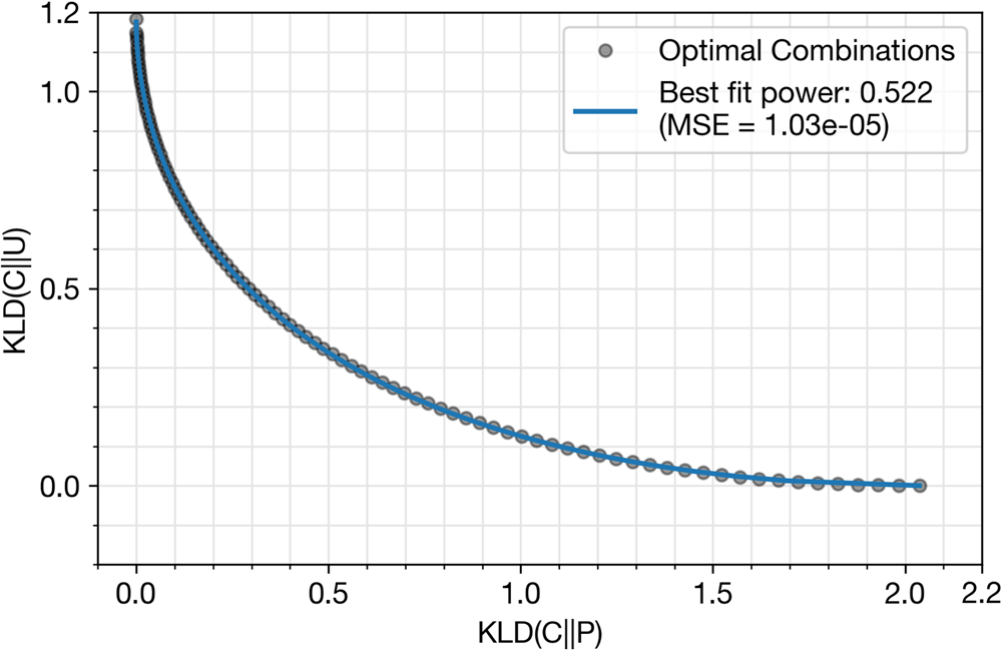
Empiric determination of the representativeness-coverage Pareto frontier. Optimal cohorts (*C*) balance KLD to the Census population (*P*) and uniform distribution (*U*). Each black circle indicates a theoretic cohort with an optimal combination of the two objectives, while the blue best-fit line plots the resultant superellipse. The superellipse power *n* and mean squared error (MSE) of the best fit line are also reported.

We use *α* = 0, 0.01, . . . , 0.99, 1 to generate the Pareto frontier of optimal solutions and find an approximate value for *n* through SciPy’s curve_fit function, yielding *n* = 0.522 (Fig. 9) and Equation (3).

3 $${\left(\frac{{\rm{KLD}}(C\,| |\,U)}{{\rm{KLD}}(P\,| | \,U)}\right)}^{0.522}+{\left(\frac{{\rm{KLD}}(C\,| | \,P)}{{\rm{KLD}}(U\,| | \,P)}\right)}^{0.522}=\,{\rm{DistOpt}}\,=1$$

We summarize the left-hand side of the equation as “distance to optimal” (DistOpt), a measure of how close a cohort is to the set of optimal solutions. It would be mathematically impossible to create a cohort with DistOpt < 1 and recruiting a cohort with an optimal DistOpt of 1 may be extremely challenging in practice. However, we may still use DistOpt as a general measure of cohort optimality that naturally balances representation and coverage. Other weightings of the two objective terms may be used by recruitment coordinators to yield cohorts that prioritize representativeness over coverage, or vice versa.

### Datasets and harmonization

U.S. Census Bureau data were obtained from July 2022 population estimates in Table “CC-EST-2022-ALL”^50^. This dataset details the United States population by the joint distribution of federal information processing standard (FIPS) code location, age (in 5-year bins), sex, race, and Hispanic origin (i.e., ethnicity) for the fifty states. We used this table because it was closest in time to *All of Us* data and provided the most granular level of population attribute detail we could find. Data from *All of Us* were obtained from the most recent snapshot available at the time of writing, Controlled Tier dataset C2022-Q4-R9. This dataset includes 413,448 unique participants with intake survey dates. We harmonized these datasets as closely as possible, but the attributes measured or options that could be selected in these datasets often differed (Table 3). Because sex is self-identified in Census data (document D-OP-GP-EN-450), we harmonized it with *All of Us* gender.

**Table 3:** Demographic attributes captured in the Census and *All of Us*, aligned by closest match

Attribute	Census Data	*All of Us* Data
Version	CC-EST-2022-ALL	C2022-Q4-R9
Age	5-year bins (0-4, 5-9,...) and 85+	Date of birth, adults only
Sex and Gender	Sex (male or female)	Gender
Race	American Indian / Alaska Native, Asian, Black, Native Hawaiian / Pacific Islander, Two or More, White	Asian, Black, Generalized Multiple Populations, Middle Eastern / North African, Native Hawaiian / Pacific Islander, White
Ethnicity	Hispanic/Latino or Non-Hispanic/Latino	Hispanic/Latino or Non-Hispanic/Latino
Location	FIPS codes	ZIP3
Imputation	Yes	No

**Fig. 10:**
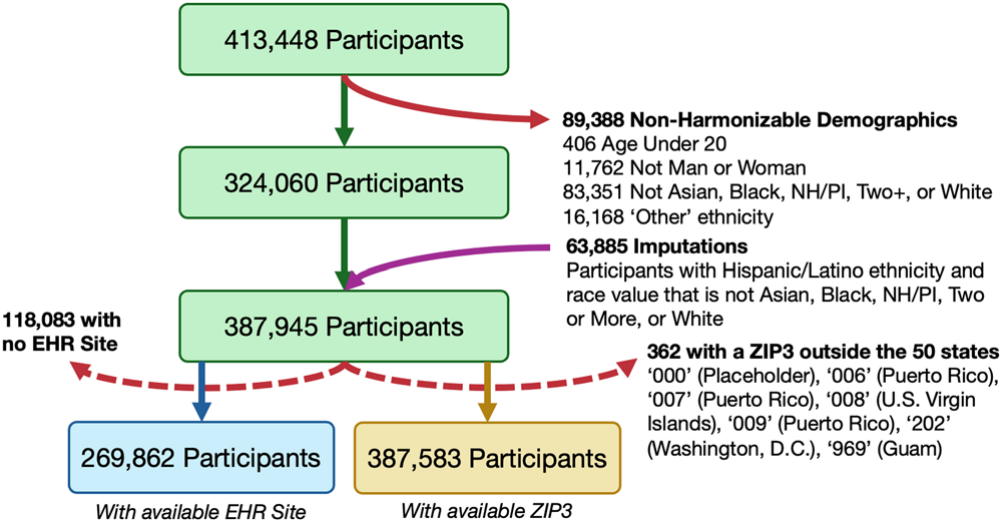
Flowchart of *All of Us* participants included in this study. Starting amounts and processing of missing information are shown in green boxes, the blue box indicates participants with available electronic health record (EHR) site code, and the yellow box indicates participants with available 3-digit ZIP code (ZIP3). Red arrows indicate exclusions and purple arrows indicate re-inclusion through imputation.

Of the *All of Us* participants, we excluded almost 90,000 because of demographics that could not be harmonized, most often missing or unknown values (Fig. 10). Upon examination of the filtered participant counts, we discovered that nearly 85% of participants who identified with Hispanic or Latino ethnicity selected a non-harmonizable race value (i.e., not Asian, Black, Native Hawaiian / Pacific Islander, Two or more races, or White). The separation of race and ethnicity questions on surveys is a known source of confusion, and the majority of Census respondents who select “Some Other Race” are of Hispanic / Latino ethnicity^51^. In the Census dataset we used, respondents who listed “Some Other Race” were assigned an imputed race value by the Census Bureau to align with the race categories shown in Table 3^52^. To align our *All of Us* data with Census counts, we applied a similar imputation strategy: participants who self-identified with Hispanic/Latino ethnicity and did not have a defined race value were assigned a random race value following the age- and gender-specific distribution of Hispanic/Latino participants who did indicate a defined race value. This imputation returned almost 64,000 participants back into the dataset (Fig. 10). Supplementary Section 1 assesses the impacts of imputation. Our final dataset restriction steps ensures that recruitment sites could be determined: a participant had to either have an available EHR site (269,862 total) for EHR site based analyses or a ZIP3 within the 50 states (387,583 total) for geographic analyses.

### Simulation methodology

**Fig. 11:**
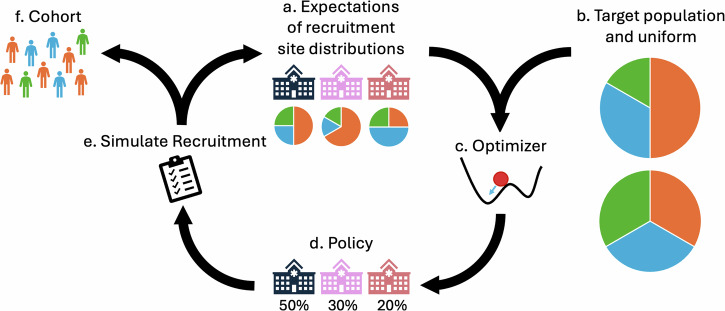
Simulation methodology flowchart for our recruitment algorithm. First, prior knowledge about response distributions at each recruitment site is initialized (**a**). Then, after defining a target population and uniform distribution (**b**), the optimizer (**c**) determines the optimal recruitment resource allocation policy (**d**) among available sites. Recruitment is then simulated (**e**), yielding participants for the cohort (**f**) and updated knowledge about site distributions (**a**). This process is iterated until some goal is reached, such as participant count or number of steps.

To evaluate our recruitment resource allocation methodology, we created a realistic yet focused simulation to mirror recruitment over time in *All of Us*. Our goal is to show how the *All of Us* cohort could have looked if our recruitment methodology had been applied, either from the cohort’s inception or from some intermediate stage of recruitment. To determine recruitment resource policies, we adapt our previously described methodology to *All of Us*^38^. Briefly, recruitment is formalized as an iterative process where resources may be allocated among sites to recruit a cohort that has some desirable characteristics like representativeness and coverage (Fig. 11). Following our prior work, iterations are defined using the maximum possible frequency of updating resource allocation. We default to quarterly resource allocation updates, equating to 21 recruitment iterations of 3 months over the span of *All of Us* recruitment from 2017 to 2022. Quarterly reporting is one of the shortest intervals used by the U.S. National Institutes of Health (NIH) in its Other Transactions^53^, so we use it as the maximum frequency of updating resource allocation. In Supplementary Section 1, we also assess our recruitment methodology using 6 annual resource allocation updates instead of 21 quarterly updates, which represents the much more common annual reporting frequency used by the NIH. Due to stochasticity, simulations are repeated 40 times for each analysis. Because some of our simulation results do not follow a normal distribution, we report 95% Bayesian credible intervals from the bayes_mvs function in SciPy^54,55^. For the same reason, we use non-parametric Wilcoxon signed-rank tests to compare simulation results to the *All of Us* historic baseline.

### Defining recruitment sites and prior knowledge

First, we determine the set of potential recruitment sites. In our experiments, we use two formulations for recruitment sites: 1) Three-digit codes for EHR sites (src_id variable), which correspond to hospital systems or 2) Three-digit ZIP codes (ZIP3), which are sometimes aggregated with nearby ZIP3s to ensure sufficient participant counts to obtain a reliable demographics distribution. There are 50 unique three-digit EHR site codes in the *All of Us* dataset compared to 386 aggregated ZIP3 sites, which were generated from 863 total ZIP3s (details in Supplementary Section 3). We restricted the *All of Us* cohort to the subset which could be recruited under a particular policy: for EHR site-based policies, this is the 269,862 participants with a defined 3-digit EHR site; for ZIP3-based policies, this is the 387,583 participants with a defined ZIP3 code in the 50 United States. To ensure our simulated cohorts are comparable to the *All of Us* cohort, our simulation recruits the same number of participants at each timestep that *All of Us* recruited, after the previous filtering step.

Next, we derive initial estimates for each site’s response distribution, the demographics of respondents who are willing to participate in the program. Knowledge about each site’s response distribution is represented through a Dirichlet distribution, which is the conjugate prior for the categorical demographics distribution. This encoding of prior knowledge allows us to ‘draw’ an estimated response distribution for each site as we update our knowledge of the sites. For EHR site-based policies, we use a non-informative Jeffreys prior with *α*_*i*_ = 0.5 ∀ *i*^56^. This choice represents a worst-case scenario where no prior knowledge of demographics at recruitment sites is available, and such information must be obtained through the recruitment process. For ZIP3-based policies, we default to the natural prior of the U.S. Census population distribution within the region.

### Adaptive recruitment resource allocation

Once prior knowledge of all sites’ response distributions has been generated, estimated categorical demographics distributions are drawn from each site’s Dirichlet. Based on these estimates, our algorithm determines the optimal allocation of resources among sites to minimize the objective function *f*, a process highlighted in Equation (4).

4 $${\rho }^{* }\leftarrow \mathop{{\rm{arg}}\,{\rm{min}}}\limits_{\rho }{\mathbb{E}}\left[\right.f(C+\rho \hat{D})\left]\right.$$

where *ρ* is a vector of fractional resource allocations at each site, *f* is an objective function, *C* is the currently recruited cohort, and $\hat{D}$ are the estimated site response distributions.

The choice of objective function *f* operationalizes recruitment goals mathematically. For instance, $f={\rm{KLD}}\left(\right.C\,| | \,P\left)\right.$ will allocate recruitment resources to minimize distance from the Census population, thereby optimizing representativeness. A combined objective like $f={\rm{KLD}}\left(\right.C\,| | \,P\left)\right.+{\rm{KLD}}\left(\right.C\,| | \,U\left)\right.$ or *f* = DistOpt from Equation (3) will optimize for a combination of both representation and coverage. To precisely control our desired combination of representativeness and coverage, we define target values for representativeness and coverage, respectively labeled KLD(*C*∣∣*P*)_*T*_ and KLD(*C*∣∣*U*)_*T*_. Then, we may optimize the recruitment policy to a cohort with the target values of representativeness and coverage by using the *L*_2_ objective function defined in Equation (5). We identify target values of representativeness and coverage by selecting a tuple on the optimal superellipse: values of KLD(*C*∣∣*P*) and KLD(*C*∣∣*U*) that satisfy Equation (3).

5 $$f=\sqrt{\left(\right.{\rm{KLD}}\left(\right.C\,| | \,P\left)\right.-{\rm{KLD}}\left(\right.C\,| | \,P{\left)\right.}_{T}{\left)\right.}^{2}+\left(\right.{\rm{KLD}}\left(\right.C\,| | \,U\left)\right.-{\rm{KLD}}\left(\right.C\,| | \,U{\left)\right.}_{T}{\left)\right.}^{2}}$$

Guided by this objective function, our algorithm identifies an optimal resource allocation among the available sites. To improve realism, we impose a constraint on the resource allocation, which limits the maximum ratio between the amount of resources allocated to the sites with the highest and lowest allocations. In our default parameter settings, we empirically set this minmax constraint to *e*^5^ ≈ 148.41, a lower ratio than is seen in reality. Such a constraint better reflects real-world recruitment at multi-site projects that typically cannot concentrate all recruitments at one site. As an added benefit, this constraint forces the algorithm to explore all sites at each recruitment step and prevents it, for instance, from missing an optimal site that joined *All of Us* later. Because the number of iterations in our simulations is often fewer than the number of sites, this method allows for efficient exploration.

### Simulated recruitment and knowledge updating

After a resource allocation is determined, we simulate participant recruitment for the iteration in a manner similar to our previous work^38^, with notable modifications to leverage the depth of historical recruitment knowledge available from *All of Us*. Most significantly, we have access to the true response distributions of recruitment sites over time because only individuals who agreed to join the program are included in our dataset. Thus, the response distribution at each site is calculated from its cumulative participant demographic distribution at the simulated timepoint in recruitment. For example, the response distribution at a site for simulated recruitment in July 2019 will be the demographics of *All of Us* participants at that site from program inception through July 2019.

First, the optimized resource allocation to each site is rounded using a floor function and the remaining sum of all fractional recruitments is assigned to the site with the highest recruitment density. Then, recruitment proceeds as a series of ‘draws’ from each site’s categorical response distribution, where the number of draws is equal to the site’s resource allocation. Each site’s recruitments constitute a new categorical likelihood distribution, which we use to update the site’s Dirichlet prior knowledge distribution, yielding a Dirichlet posterior. For ZIP3-based sites using a Census prior, this prior is overwritten and the Dirichlet demographic distribution is set to the actual recruitments from the site. Groups which were not recruited within the first iteration (i.e., count of 0) have their *α* values set to a small positive number of 1 × 10^−10^. This process is repeated for every recruitment iteration, with response distribution knowledge improving at each step.

## Supplementary information

Supplementary Information

## Data Availability

*All of Us* Research Program data (dataset C2022-Q4-R9) are available through a data use agreement in the Researcher Workbench. *All of Us* Registered Tier and Controlled Tier data are available to researchers affiliated with a participating institution, pursuant to the program's policies. EHR site based results can be reproduced with Registered Tier data, while ZIP3 based results require Controlled Tier access. *All of Us* notes that participant counts may differ between data tiers due to different curation processes. Under the program's Data User Code of Conduct, these data cannot be published or redistributed.

## Code availability

The underlying code for data collection and analysis is available on GitHub and can be accessed at https://github.com/vaborza/aourp_paper. The GitHub page also includes instructions for replication analyses, provided that the user can set up an appropriate validation workspace in *All of Us*.
