# Supplementary material for: Computational strategic recruitment for representation and coverage studied in the All of Us Research Program
Source: NPJ Digit Med. 2025 Jul 3;8:402. doi: 10.1038/s41746-025-01804-x (PMC12229505; doi:10.1038/s41746-025-01804-x)
Supplement: Supplementary file 1 — Supplementary Information [file 41746_2025_1804_MOESM1_ESM.pdf]

Supplementary Information

1 Alternative simulation parameters

First, we re-introduce participants without a defined EHR site into our simulated cohorts and *All of Us* comparisons at each timestep. These participants represent a significant (30.44%) yet unmodifiable component of our recruitment strategy, as there is no clear parametrization of the recruitment sites they join *All of Us* from. Including these participants slightly skews both the historical *All of Us* cohort and our simulation cohorts towards Census-representativeness and away from coverage, but our simulation cohorts still improve upon historical *All of Us* results (Supplementary Table 1). We also consider the impact of tightening the minmax policy constraint; as expected, stricter constraints on site-to-site differences in resource allocation limit the algorithm's ability to improve upon the representativeness and coverage of the final cohort, but our algorithm still improves upon historical *All of Us* results. Despite substantial differences in $\mathrm{KLD}(C||U)$, the more constrained algorithm generates a final cohort with equal proportion of participants underrepresented by race/ethnicity.

The final simulation parameter we modified was the number of recruitment iterations. Our default experiment assumed a new recruitment policy could be developed every 3 months, for a total of 21 iterations; we also analyzed if the recruitment policy could only be updated annually, for a total of 6 iterations. For a large scale program like *All of Us*, this may be a more realistic timeline for re-allocating funding among sites. Annual recruitment policy updates yielded a final cohort that was generally similar to quarterly updates, but with lower Census-representativeness and a higher proportion of underrepresented participants. The confidence intervals for all final cohort measures were wider for 1-year policy updates compared to 3-month policy updates, indicating that a broader range of final cohort demographics were recruited. Although annual policy updates led to somewhat less control over the final cohort, they still provided significant improvements in representativeness and coverage over the historical *All of Us* cohorts. A two-sided Wilcoxon signed-rank test yielded $M = -10$ and $p = 0.00652$ for representativeness and $M = -20$ and $p = 1.819 \times 10^{-12}$ for coverage.

Because our selection of a recruitment goal was empirically derived based on historical *All of Us* recruitment, we also investigated how our simulated cohorts would look under different prioritizations of representativeness and coverage. Building off the Pareto frontier for recruitment that we derived in Figure 5, we assessed the extreme examples where only representativeness or coverage were used as the objective function. Unsurprisingly, the Census-representativeness objective function, $\mathrm{KLD}(C||P)$, yielded the lowest proportion of underrepresented participants of all our experiments, while the coverage objective function, $\mathrm{KLD}(C||U)$, yielded the highest. Notably, over 50% of participants in the final simulated cohort for the coverage objective function were considered UBR by race or ethnicity. This reinforces the substantial site-to-site demographic variations that we see and, thus, highlights the value of strategic recruitment.

In addition to the *All of Us* historic baseline, we analyzed a uniform resource allocation policy that assigns an equal amount of recruitment resources to each available

site each quarter. The uniform policy yields a slightly more covering but substantially less representative final cohort than the historic *All of Us* baseline. A two-sided Mann-Whitney U test between our proposed methodology and the uniform allocation policy yielded a U value of 0 and p-value of 1.435×10^{-14} for both final representativeness and coverage. Thus, strategic resource allocation yields a significantly more representative and covering cohort than a uniform allocation baseline.

Another factor that may influence our simulation results was the imputation of race for individuals who identify as Hispanic/Latino and did not list a defined race value. While this process is similar to the imputation processes used by the U.S. Census Bureau to establish the true population baseline, it is inherently a stochastic process. Thus, sites may end up with slightly different racial distributions among Hispanic/Latino participants from one imputation to another. To evaluate the impact of imputation variations on our results, we repeated the imputation process five additional times using different random seeds, generating five additional historic *All of Us* cohorts. For each of these five historic cohorts, we repeated our recruitment simulation experiments 40 times and analyzed the final $\text{KLD}(C||P)$ and $\text{KLD}(C||U)$ values. Varying imputations had minimal impact on final simulated cohort representativeness and coverage (Supplementary Table 2). Inter-experimental variation was 1-2 orders of magnitude less than the effect of computational recruitment.

2 Site participants and Asian representation

To further investigate the decreasing representation of Asian-identifying participants in *All of Us* over time (Figure 1), we studied the proportion of cumulative *All of Us* participants that each recruitment site contributed over time. We then compare these proportions to each site's final total representation of Asian participants (Supplementary Figure 1). Sites that had a higher representation of Asian participants than the Census (blue coloration) contributed steadily to *All of Us* totals, around 10% of cumulative participants. On the other hand, sites with substantial underrepresentation of Asian participants (deep red coloration) contributed relatively more participants to *All of Us* over time.

3 Geographically-defined recruitment policies

The most substantial change we make to our simulation is modifying the policy space to allocate recruitment resources to geographic regions instead of EHR sites. First, we identify the subset of *All of Us* participants with a defined ZIP3 within the 50 United States for comparison with Census data. This yields 387,583 participants, more than the 269,862 participants with a defined EHR site (Figure 9). The *All of Us* final cohort demographics for this subset of participants are detailed in Supplementary Table 3 and closely mirror the total final *All of Us* cohort. We first define geographic regions using the first three digits of a ZIP code (ZIP3), the finest location granularity available in *All of Us*. This process yields 863 ZIP3s with at least 1 *All of Us* participant.

To decrease variance in our geographic distributions, we combine ZIP3s with less than 100 participants together to generate geographic recruitment sites, each with at least 100 participants. We manually aggregated ZIP3s according to four preferences:

1) when possible, aggregate ZIP3s within the same state; 2) aggregate adjacent regions together, preferring larger shared borders; 3) aggregate urban areas with surrounding suburbs when aggregation is needed; and 4) minimize the total number of generalizations needed. The aggregation process yields 386 geographic recruitment sites (Supplementary Table 4) and their geographic distribution is plotted in Supplementary Figure 2. The only geographic recruitment site with less than 100 participants was the aggregated state of Alaska, which had 97 participants and no neighboring ZIP3s to combine with. Although the geographic formulation of recruitment sites is somewhat less practical to implement than the EHR-site-based formulation, it provides insight into how the size of the policy space (i.e., number of sites) can affect recruitment strategies.

By widening the policy space from 50 EHR sites to 386 geographic sites, our algorithm generates even more representative and covering cohorts which more closely approach the theoretic Pareto frontier (Supplementary Figure 3). When recruitment simulations are initialized identically using an uninformed Jeffreys prior for each site and a target point of KLD$(C||P) = 0.04$ and KLD$(C||U) = 0.909$, a ZIP3-defined policy space achieves a mean final KLD$(C||P)$ of 0.0976 [0.0968 - 0.0984] and KLD$(C||U)$ of 0.9311 [0.9306 - 0.9316]. The closest comparison in the EHR site-defined policy space is the simulation which includes participants without an EHR site, yielding a mean final cohort KLD$(C||P)$ of 0.1406 [0.1398 - 0.1413] and KLD$(C||U)$ of 0.9586 [0.9578 - 0.9594]. Utilizing Census demographic distributions of ZIP3 sites as priors did not improve final cohort representation or coverage compared to the uninformed Jeffreys prior, with mean final cohort KLD$(C||P)$ of 0.0994 [0.0987 - 0.1001] and KLD$(C||U)$ of 0.9317 [0.9312 - 0.9321].

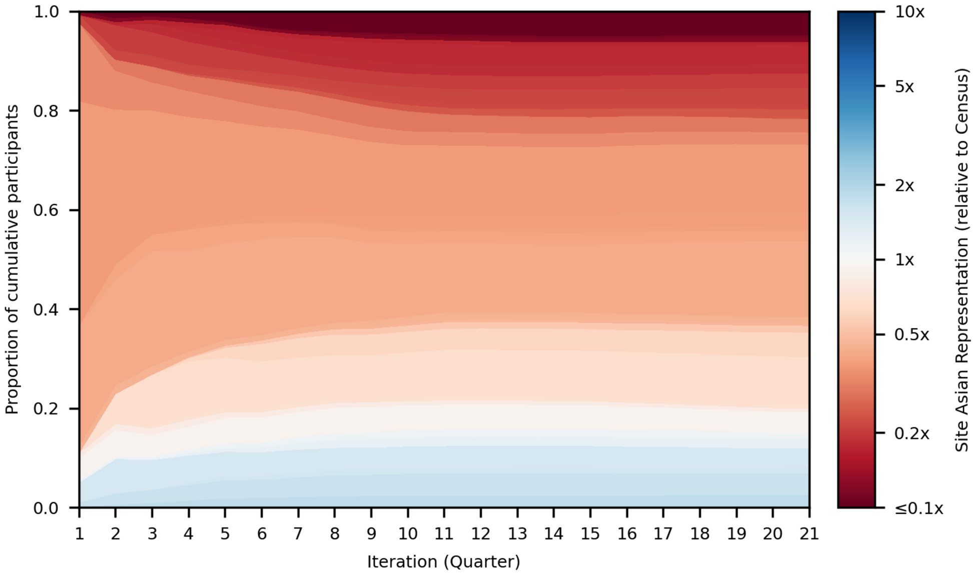

Supplementary Figure 1 Contributions of recruitment sites to the cumulative participant total of *All of Us* over time. Each site is represented by a vertical band, where the height of the band indicates what proportion of *All of Us*'s cumulative participant total originated from that site at each quarter. Sites are colored by their overall representation of Asian individuals, relative to Census levels, with blue colors indicating overrepresentation, white indicating Census-equal representation, and red colors indicating underrepresentation.

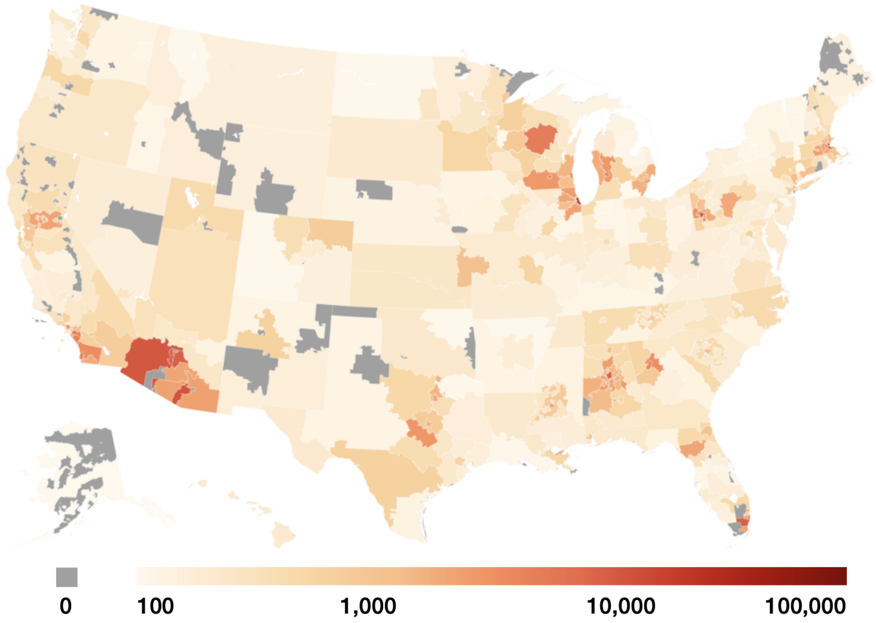

Supplementary Figure 2 Geographic distribution of participants in *All of Us*. Aggregated geographic regions are colored by the number of participants from that region, with darker colors indicating more participants. Gray regions indicate ZIP codes that could not be matched or geographic regions with no *All of Us* participants.

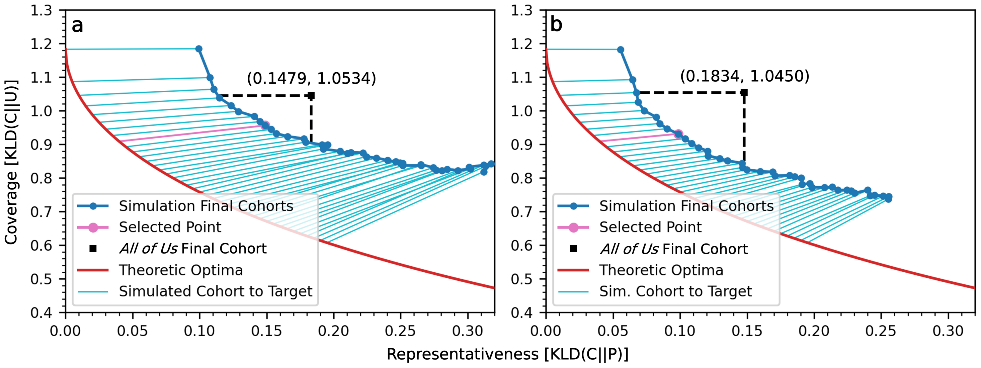

Supplementary Figure 3 Comparison of the simulated final cohorts for ZIP3 and EHR defined sites. Simulated final cohorts are represented with dark blue dots and lines with ZIP3-defined sites shown in panel a and EHR-defined sites shown in panel b. The representativeness-coverage Pareto optimal frontier is shown with a red line. Defining recruitment policies over ZIP3 sites allows for greater improvements in both coverage and representativeness compared to EHR sites, evidenced by the closer proximity to the theoretic Pareto frontier line. The final *All of Us* cohorts for participants with a defined ZIP3 and defined EHR site are shown as black squares in the corresponding panels. Cyan lines show the relationship between a cohort's final representativeness, coverage, and target point while the pink line and outlined point indicate the target point of (0.04, 0.909).

Supplementary Table 1 Effects of simulation parameters on final cohort representativeness, coverage, and proportion of underrepresented participants. We include two *All of Us* historical cohorts (both total and limited to those with an EHR site) as well as *All of Us*'s goals as baselines. The 'Modification' column describes which parameter we modified in simulation to yield the results in the other columns. All results are shown as mean [95% CI] across 40 experimental replicates.

Modification	Final KLD($C\|\|P$)	Final KLD($C\|\|U$)	% Underrep.	% Underrep. by race/ethnicity
All of Us Goal	—	—	75	45
All of Us w/ Site	0.1834	1.0450	69.42	45.15
All of Us Total	0.1480	1.0528	66.58	43.61
Our Simulation (Default)	0.1508 [0.1496-0.1520]	0.9557 [0.9550-0.9564]	68.20 [68.10-68.31]	46.71 [46.65-46.77]
Including non-site participants	0.1406 [0.1398-0.1413]	0.9586 [0.9578-0.9594]	67.49 [67.42-67.57]	46.58 [46.53-46.63]
Tighten minmax constraint to e^4	0.1557 [0.1548-0.1566]	0.9578 [0.9573-0.9584]	68.35 [68.27-68.43]	46.78 [46.72-46.84]
1-year iterations	0.1790 [0.1754-0.1826]	0.9621 [0.9604-0.9639]	69.79 [69.57-70.01]	48.57 [48.34-48.79]
KLD($C\|\|P$) Objective	0.0979 [0.0971-0.0987]	1.1964 [1.1952-1.1975]	58.51 [58.43-58.59]	34.50 [34.44-34.57]
KLD($C\|\|U$) Objective	0.5106 [0.5073-0.5139]	0.8562 [0.8535-0.8590]	77.82 [77.55-78.09]	64.05 [63.83-64.27]
Uniform Allocation	0.2666 [0.2662-0.2669]	1.0412 [1.0405-1.0420]	71.34 [71.33-71.36]	0.5157 [51.55-51.60]

Supplementary Table 2 Effects of imputation variations on final cohort representativeness and coverage. Because varying imputation affects both the historic *All of Us* cohort and the resultant simulated cohorts, we present representativeness and coverage values for both historic and simulated cohorts. Simulation results are shown as mean [95% CI] across 40 experimental replicates.

Imputation Seed	Simulated KLD($C\|\|P$)	Historic KLD($C\|\|P$)	Simulated KLD($C\|\|U$)	Historic KLD($C\|\|U$)
8080	0.1508 [0.1496-0.1520]	0.1834	0.9557 [0.9550-0.9564]	1.0450
8081	0.1513 [0.1505-0.1522]	0.1831	0.9547 [0.9539-0.9554]	1.0458
8082	0.1482 [0.1471-0.1492]	0.1830	0.9542 [0.9536-0.9549]	1.0465
8083	0.1493 [0.1481-0.1504]	0.1826	0.9555 [0.9548-0.9561]	1.0465
8084	0.1519 [0.1512-0.1527]	0.1827	0.9557 [0.9551-0.9563]	1.0460
8085	0.1502 [0.1492-0.1513]	0.1828	0.9548 [0.9541-0.9556]	1.0457

Supplementary Table 3 Participant demographics in *All of Us*. ZIP3 refers to 3-digit ZIP code.

Demographic Group	*All of Us* Count	*All of Us* Count with ZIP3 (%)	Census Count (%)
Age			
20-44	126,540 (32.62)	126,414 (32.61)	109,958,279 (44.27)
45-64	139,955 (36.08)	139,806 (36.07)	81,287,170 (32.73)
65+	121,450 (31.31)	121,363 (31.31)	57,115,071 (23.00)
Gender			
Female	239,771 (61.81)	239,571 (61.81)	126,509,045 (50.94)
Male	148,174 (38.19)	148,012 (38.19)	121,851,475 (49.06)
Race			
Asian	16,756 (4.32)	16,742 (4.32)	16,155,290 (6.50)
Black	86,781 (22.37)	86,686 (22.37)	32,828,357 (13.22)
Native Hawaiian / Pacific Islander	872 (0.22)	872 (0.22)	607,035 (0.24)
Two or More Races / Mixed Race	13,533 (3.49)	13,507 (3.48)	5,312,304 (2.14)
White	270,003 (69.60)	269,776 (69.60)	193,457,534 (77.89)
Ethnicity			
Hispanic / Latino	72,917 (18.80)	72,729 (18.76)	41,378,366 (16.66)
Non-Hispanic / Latino	315,028 (81.20)	314,854 (81.24)	206,982,154 (83.34)

Supplementary Table 4: Three-digit ZIP codes (ZIP3s) of participants in *All of Us*. ZIP3s with fewer than 100 participants were aggregated as shown below. ZIP3s corresponding to regions outside the 50 United States are denoted with "[excl.]" and were excluded from geographic analyses.

	Unaggregated ZIP3s	
986	985	984
983	982	981
980	973	969 [excl.]
959	958	957
956	954	953
952	951	950
949	947	946
943	941	939
935	934	933
932	928	927
926	925	922
921	920	919
918	917	916
915	914	913
912	911	910
908	907	906
902	900	891
890	863	859
857	856	855
853	852	851
850	802	801
800	778	774
773	767	766
765	751	750
704	701	700
611	610	608
607	606	605
604	603	602
601	600	559

Continued on the next page

Supplementary Table 4: Three-digit ZIP codes (ZIP3s) of participants in *All of Us*. ZIP3s with fewer than 100 participants were aggregated as shown below. ZIP3s corresponding to regions outside the 50 United States are denoted with “[excl.]” and were excluded from geographic analyses.

558	557	554
551	550	549
548	547	546
545	544	540
539	537	534
532	531	530
496	495	494
493	492	483
482	481	480
462	441	392
391	390	379
378	372	368
367	366	365
362	361	360
359	358	357
356	355	354
352	351	350
347	346	344
342	341	339
337	336	335
334	333	331
330	329	328
327	326	323
322	321	320
306	305	303
302	300	292
291	277	276
223	222	220
212	209	208
202 [excl.]	201	194

Continued on the next page

Supplementary Table 4: Three-digit ZIP codes (ZIP3s) of participants in *All of Us*. ZIP3s with fewer than 100 participants were aggregated as shown below. ZIP3s corresponding to regions outside the 50 United States are denoted with "[excl.]" and were excluded from geographic analyses.

193	191	165
164	163	162
161	160	159
157	156	155
154	153	152
151	150	126
116	115	114
112	111	109
104	103	101
100	076	067
062	029	028
027	026	025
024	023	022
021	020	019
018	017	016
015	014	012
009 [excl.]	008 [excl.]	007 [excl.]
006 [excl.]	000 [excl.]	
	Aggregated ZIP3s	
995-999	988, 989, 991, 993, 994	990, 992
970-972	974-979	967, 968
903, 904, 905	923, 924	930, 931
936, 937	940, 944	945, 948
955, 960, 961	894, 895, 897, 898	870, 871, 873, 874
875, 877	880-883	840, 841, 843, 844
845-847, 860, 864, 865	832, 833, 835, 838	836, 837
820, 822, 824-829, 830	803-805	806, 807
808-810	811-816	752, 753
754, 755	756-759	760-764
768, 769, 790-797	775-777	770, 772

Continued on the next page

Supplementary Table 4: Three-digit ZIP codes (ZIP3s) of participants in *All of Us*. ZIP3s with fewer than 100 participants were aggregated as shown below. ZIP3s corresponding to regions outside the 50 United States are denoted with "[excl.]" and were excluded from geographic analyses.

779, 780-782, 788	783-785	786, 787, 789
798, 799	730, 731, 734-739, 745, 747, 748	740, 741, 743, 744, 746
716-719, 727-729	720-722	723-726
703, 705, 706	707, 708	711-714
680, 681	683-689, 691, 693	664-669, 674, 676, 677
670-673, 675, 678, 679	630, 631, 633	634, 635, 644-646, 650-653
636-639, 647, 648, 654-658	640, 641, 660-662	609, 617
612-614, 615, 616	618, 619, 623-627	620, 622, 628, 629
590-597	598, 599	580, 581
582-588	570-577	553, 560-562
563, 564	565-567	535, 538
541-543	500-503, 508	504-507, 510-515
520, 526-528	521-525	484, 485
486, 487	488, 489	490, 491
497-499	460, 479	461, 478
463, 464	465, 466	467-469, 473
470-472, 474-477	430-433	434-436, 440, 448, 449
437-439, 446, 447, 457	442-445	453-455, 458
400-402	403-406	407-409, 412, 413, 415, 416, 418, 420-427
410, 411, 450-452, 456	386-389	393, 397
394-396	370, 371, 384, 385	373, 374
376, 377	380-383	363, 364
324, 325	338, 349	304, 308, 309, 313, 314
301, 307	310, 312	315-319, 398
290, 298, 299	293, 296, 297	294, 295
270-274	275, 278, 279, 285	280-282
283, 284	286-289	250-259, 260-268
221, 224, 225	226-229	230-232, 238, 239
233-237	240-249	206, 207

Continued on the next page

Supplementary Table 4: Three-digit ZIP codes (ZIP3s) of participants in *All of Us*. ZIP3s with fewer than 100 participants were aggregated as shown below. ZIP3s corresponding to regions outside the 50 United States are denoted with "[excl.]" and were excluded from geographic analyses.

210, 211, 214	215, 217	200, 203
197-199, 216, 218, 219	158, 167, 169	166, 168
170-172	173-176	177, 178
179, 180-182, 195, 196	183-188	189, 190
143-146	140-142, 147	137-139, 148, 149
130-132	128, 133-136	124, 125, 127
120-123	117-119	110, 113
107, 108	105, 106	088, 089
085, 086	080-083	077, 087
078, 079	074, 075	070-073
068, 069	064-066	060, 061
054, 056, 058, 129	050-053, 057	044, 046, 047, 049
043, 045, 048	040-042	035, 038, 039
032, 037	030, 031, 033, 034	010, 011, 013
